# A metagenomic analysis for combination therapy of multiple classes of antibiotics on the prevention of the spread of antibiotic-resistant genes

**DOI:** 10.1080/19490976.2023.2271150

**Published:** 2023-10-31

**Authors:** Matthew Igo, Lei Xu, Ashok Krishna, Sharron Stewart, Lin Xu, Zhihua Li, James L. Weaver, Heather Stone, Leonard Sacks, Timothy Bensman, Jeffry Florian, Rodney Rouse, Xiaomei Han

**Affiliations:** a Division of Applied Regulatory Science, Office of Clinical Pharmacology, Office of Translational Sciences, Center for Drug Evaluation and Research, U. S. Food and Drug Administration, Silver Spring, MD, USA; b Office of Medical Policy, Center for Drug Evaluation and Research, U. S. Food and Drug Administration, Silver Spring, MD, USA; c Division of Infectious Disease Pharmacology, Office of Clinical Pharmacology, Office of Translational Sciences, Center for Drug Evaluation and Research, U. S. Food and Drug Administration, Silver Spring, MD, USA

**Keywords:** Metagenomic analysis, combination therapy, antibiotics, antibiotic-resistant genes

## Abstract

Antibiotics used systemically to treat infections may have off-target effects on the gut microbiome, potentially resulting in the emergence of drug-resistant bacteria or selection of pathogenic species. These organisms may present a risk to the host and spread to the environment with a risk of transmission in the community. To investigate the risk of emergent antibiotic resistance in the gut microbiome following systemic treatment with antibiotics, this metagenomic analysis project used next-generation sequencing, a custom-built metagenomics pipeline, and differential abundance analysis to study the effect of antibiotics (ampicillin, ciprofloxacin, and fosfomycin) in monotherapy and different combinations at high and low doses, to determine the effect on resistome and taxonomic composition in the gut of Balb/c mice. The results showed that low-dose monotherapy treatments showed little change in microbiome composition but did show an increase in expression of many antibiotic-resistant genes (ARGs) posttreatment. Dual combination treatments allowed the emergence of some conditionally pathogenic bacteria and some increase in the abundance of ARGs despite a general decrease in microbiota diversity. Triple combination treatment was the most successful in inhibiting emergence of relevant opportunistic pathogens and completely suppressed all ARGs after 72 h of treatment. The relative abundances of mobile genetic elements that can enhance transmission of antibiotic resistance either decreased or remained the same for combination therapy while increasing for low-dose monotherapy. Combination therapy prevented the emergence of ARGs and decreased bacterial diversity, while low-dose monotherapy treatment increased ARGs and did not greatly change bacterial diversity.

## Introduction

The spread of antibiotic-resistant bacteria is a global problem, resulting in less effective treatment options for bacterial infections and greater morbidity and mortality from infectious diseases.^1,2^ There is an increasing threat of infections from multidrug-resistant (MDR) bacteria with approximate 1.27 million deaths caused by MDR bacteria in 2019.^3,4^ The selective pressure from antibiotics may result in the emergence of bacteria that contain antibiotic-resistant genes (ARGs) that may become the predominant pathogens.^5^ It has been shown that the use of antibiotics promotes the transfer of genetic material and potentially antibiotic resistance through mobile genetic elements (MGEs) such as plasmids,^6^ and other elements such as transposons and integrons.^7–9^ With few new antibiotics being developed, strategies to preserve the efficacy of current antibiotics need to be explored.^10^

One approach to prevent the spread of ARGs is combining different classes of antibiotics, known as combination therapy.^11^ In theory, combination therapy with antibiotics with different mechanisms of action would show increased effectiveness by attacking microbial viability at multiple different points and diminishing compensatory ability. The efficacy of combination therapy for the treatment of bacterial infections is controversial, as studies on the differences between mono- and combination therapy are scarce;^12,13^ however, several clinical studies have found that combination therapy has prevented the rise of antibiotic resistance when treating infections.^14,15^ The diverse mechanisms of action when using a combination of antibiotics has also been shown to be successful in treating MDR organisms,^16–19^ and studies have suggested that the synergistic effect of combinations of antibiotics may allow for the use of lower dosages of multiple antibiotics to treat infections.^20–22^

In prior experiments, we used a hollow-fiber model^23^ to demonstrate that combinations of antibiotics delayed or reduced the emergence of antimicrobial resistance in a suspension of *Escherichia coli* CFT073 compared to treatment with single agents. These findings suggested that the use of combinations of antibiotics in clinical practice may be helpful to reduce the emergence of bacterial resistance in common gram-negative infections, such as cystitis.

Previously, our group tested single antibiotics in a mouse model of urinary tract infections at doses equivalent to those commonly used for treatment of patients.^1^ The antibiotics tested were ampicillin from the beta-lactam class, ciprofloxacin from the fluoroquinolone class, and fosfomycin from the phosphonic acid class. That research revealed that using standard monotherapy treatment caused an increase in potentially pathogenic bacteria in the gut, a decrease in overall gut microbiome diversity, and an increase in ARG abundance.^1^

In the present study, a metagenomic approach was used to examine the effects of single low-dose antibiotics and high and low doses of combinations of antibiotics on the gut microbiome of BALB/c mice. Metagenomics allows for the analysis of microorganisms at a community level based on species-level abundance changes^24,25^ and the resistome through analysis of ARG abundance over time.^26–28^ Shotgun metagenomic and 16S RNA data have allowed for deeper understanding of microbial population abundance and diversity and helped identify the factors that can influence community populations.^29,30^ Publicly available tools such as Metaphlan3^31^ and QIIME,^32^ and the decreasing cost of genomic sequencing^33^ have allowed for greater research to be conducted into metagenomics and microbial communities, including the analysis of MDR organisms.

## Materials/methods

### Experimental design

#### Animal model and antibiotic treatment

All mice used in this research were 8–10-week-old female Balb/c mice, which were housed and cared for according to the Guide for the Care and Use of Laboratory Animals 8th Edition, under an Institutional Animal Care and Use Committee approved protocol in the AAALAC accredited Animal Program of the White Oak Federal Research Center. All mice in a treatment group were group housed and provided food and water ad libitum. Treatment groups were created for different combinations of the same three classes of antibiotics used in the original study (Table 1), represented by ampicillin, ciprofloxacin, and fosfomycin. Treatment groups were (1) triple combination of ampicillin, ciprofloxacin, and fosfomycin, and dual combinations of (2) ampicillin and ciprofloxacin, (3) ampicillin and fosfomycin, and (4) ciprofloxacin and fosfomycin all at a high and a low concentration, and monotherapy treatments of (5) ciprofloxacin, (6) fosfomycin, and (7) ampicillin at a low concentration (higher concentration monotherapies were reported in the earlier publication.^1^ Two types of control mouse groups were used: a naïve control and excipient control. Naïve control mice were given no antibiotics and no antibiotic excipients, whereas excipient control groups received only the antibiotic excipient and not the antibiotic. For the combination treatment groups, excipient control group mice were given excipient combinations corresponding to excipients of the combination treatment group. Ampicillin trihydrate (Sigma, St. Louis, MO), cipro 5% suspension (Bayer HealthCare Pharmaceuticals Inc., Wayne, NJ), or monurol (fosfomycin tromethamine) (Forest Pharmaceuticals Inc., St. Louis, MO, USA) were used for the treatment of animals. Ampicillin was dissolved in 0.1 M HCl, ciprofloxacin (microgranules: hypromellose 3 cP, magnesium stearate, polyacrylate dispersion 30%, polysorbate 20, and povidone 25) was dissolved in ciprofloxacin excipient (diluent: medium chain triglycerides, purified water, soy-lecithin, strawberry flavor 52312 and 54267, sucrose micronized), and fosfomycin (Monurol) was dissolved in water to create oral dosing solutions. Antibiotic doses were determined using in-house pharmacokinetic models to achieve a high concentration of antibiotics in the urinary bladder based on previous published results^34^ from studies on the development of antibiotic resistance in *Escherichia coli*. Dosages that were considered as the “high” concentration treatment levels were 200 mg/kg body weight of ampicillin, 50 mg/kg body weight of ciprofloxacin, and 1000 mg/kg of fosfomycin. “Low”-dose concentrations were created to assess whether suboptimal exposure might change microbiome diversity and ARG profile. Low doses were 1/10th of the higher dose concentration. Both high and low doses of ampicillin and ciprofloxacin were administered twice daily, 8 h apart, while fosfomycin was administered once daily. All treatments were performed for 3 d. Mice from each treatment group were sacrificed after 24, 48, and 72 h of treatment.

**Table 1.** A–C: experimental design for all antibiotic treatments and dosage.

A. Experimental Design of All Cohorts							
Combination Treatment	High Dose^1^	Low Dose^2^					
Triple Combination	**X**	**X**					
Ampicillin and Ciprofloxacin	**X**	**X**					
Ampicillin and Fosfomycin	**X**	**3**					
Ciprofloxacin and Fosfomycin	**X**	**3**					
Ampicillin	**X^4^**	**3**					
Ciprofloxacin	**X^4^**	**X**					
Fosfomycin	**X^4^**	**X**					
B. Treatment Groups							
Cohorts	Naïve Control	Excipient Control	Treatments				
Triple Combination; High Dose	*n* = 3	*n* = 3	*n* = 8				
Triple Combination; Low Dose	*n* = 5	*n* = 6	*n* = 14				
Ampicillin and Ciprofloxacin; High Dose	*n* = 2	*n* = 6	*n* = 18				
Ampicillin and Ciprofloxacin; Low Dose	*n* = 6	*n* = 6	*n *= 18				
Ampicillin and Fosfomycin; High Dose	*n* = 5	*n* = 4	*n* = 17				
Ciprofloxacin and Fosfomycin; High Dose	*n* = 1	*n* = 2	*n* = 8				
Ciprofloxacin; Low Dose	*n* = 0	*n* = 4	*n* = 9				
Fosfomycin; Low Dose	*n* = 0	*n* = 6	*n* = 18				
C. Antibiotic Treatment Times							
Ampicillin at 200 mg/kg for high dose and 20 mg/kg for low dose							
Date	Monday	Tuesday	Wednesday		Thursday		Friday
Procedure	Animal sacrifice	AM oral	AM oral	Animal sacrifice	AM oral	Animal sacrifice	Animal sacrifice
	for controls	8 h interval	8-h interval	for 24-h treatment	8-h interval	for 48-h treatment	for 72-h treatment
		PM oral	PM oral		PM oral		
Group	Naïve controls	All but control	All but control		All but control		All mice
Ciprofloxacin at 50 mg/kg for high dose and 5 mg/kg for low dose							
Date	Monday	Tuesday	Wednesday		Thursday		Friday
Procedure	Animal sacrifice	AM oral	AM oral	Animal sacrifice	AM oral	Animal sacrifice	Animal sacrifice
	for controls	8-h interval	8-h interval	for 24-h treatment	8-h interval	for 48-h treatment	for 72-h treatment
		PM oral	PM oral		PM oral		
Group	Naïve controls	All but control	All but control		All but control		All mice
Fosfomycin at 1000 mg/kg for high dose and 100 mg/kg for low dose							
Date	Monday	Tuesday	Wednesday		Thursday		Friday
Procedure	Animal sacrifice	AM oral	AM oral	Animal sacrifice	AM oral	Animal sacrifice	Animal sacrifice
	for controls			for 24-h treatment		for 48-h treatment	for 72-h treatment
Group	Naïve controls	All but control	All but control		All but control		All mice

### Extraction and processing of genomic material

After 24, 48, or 72 h of treatment, stool samples were obtained from the distal ileum and proximal colon of the intestinal tract at the time of sacrifice of mice from each treatment group. Genomic DNA was extracted using QIAamp DNA Stool Mini kit (Qiagen, MD, USA), modified in the first step using Tungsten Carbide Beads 3 mm (Qiagen, Str. 140724 Hilden, Germany) in sample disruption with TissueLyser LT (Qiagen, MD, USA) for high-speed shaking at 50 Hz for 1 min. Stool samples were then homogenized in lysis buffer and heated for 10 min at 70°C, and PCR inhibitors were removed by inhibitEX Tablets (Qiagen, MD, USA). The supernatants were enzymatically digested using proteinase K (Qiagen, MD, USA) for 5 min at 70°C. Genomic DNA was purified using QIAamp Mini Spin Column. DNA quality was evaluated with an Agilent 2100 Bioanalyzer A260/280 and quantified with a Qubit 4 Fluorometer (Thermo Fisher Scientific, NY, USA). Genomic DNA libraries were created by either one of the following approaches:

1. Using 50 ng of genomic DNA for library preparation following the Nextera DNA Library Prep Reference Guide 2016. Tagmented DNA was purified using DNA Clean & ConcentractorTM-5 (Zymo Research, Irvine, CA, USA), and library DNA was purified using AMPure XP beads (Beckman Coulter Life Science, Brea, CA, USA)
2. Using 1–50 ng of genomic DNA for library preparation following the Nextera DNA Flex Library Prep reference guide 2019. Tagmented DNA was created by using bead-linked transposomes. The DNA library was purified using sample purification beads provided by Illumina. Incubation and thermal cycling were performed using a Thermo Scientific Arktik Thermal Cycler (Thermo Fisher Scientific, Waltham, MA, USA).

The size range was assessed on the Agilent 2100 bioanalyzer (Agilent Technology, Santa Clara, CA, USA) with High Sensitivity DNA Analysis Kit (Agilent Technology, Santa Clara, CA, USA) and quantified on the Qubit 4 Fluorometer. Library pools for sequencing were created according to the NextSeq System Denature and Dilute Libraries Guide (Illumina, 2016). Metagenome sequencing was performed using the NextSeq 500 sequencing system ((Illumina, San Diego, CA, USA) with paired-end shotgun sequencing.^35^ Not all animals yielded usable sequencing data due to premature loss of the animal, limited stool, or insufficient amount of extracted DNA. The treatment groups, group sizes, and treatment schedules are shown in Table 1. The samples were never pooled in any of the experiments.

### Data quality control

Illumina bcl2fastq version 2.18.0.12 (RRID:SCR_015058, Illumina) was used to demultiplex and trim adapter sequences. The quality of the shotgun metagenomic sequences was determined using FastQC version 0.11.8,^36^ to detect possible quality control issues. Trimmomatic version 0.39 was used to trim low-quality reads.^37^ Trimmomatic parameters were set to SLIDINGWINDOW 4:15 and MINLEN 30. Mouse genomic DNA was removed from microbiome samples by aligning reads to the mouse genome database GRCm39 with Burrows-Wheeler alignment (BWA) tool v. 0.7.16.^38^ Unmapped reads were extracted using SAMtools^39^ to create SAM files containing bacterial DNA. SAM files were converted to FASTQ files using the samtoFASTq function in Picard Tools version 2.1.1,^40^ and then to FASTA files using bash scripting code. The sample information and read counts at different stages of the process are shown in Supplementary Table S1. Metagenome sequencing data were deposited in the National Center for Biotechnology Information (NCBI) BioSample Submission Portal under Bioproject PRJNA886985.

### Mapping of ARGs and MGEs

A custom BLAST database was created using the makeblastdb function in the NCBI BLAST+ tool^41^ from the Comprehensive Antibiotic Resistance Database protein homolog model version 3.0.3 (CARD)^42^ FASTA file, and blastx command was used to characterize the resistome by mapping reads against this custom BLAST database. The results were exported to a custom SQL database and hits that fit the criteria of an e-value ≤1e-5, pindet ≥ 80, and qcovs ≥ 50 were counted and summed. MGE counts were determined by mapping bacterial reads against MobileGeneticElementDatabase^43^ using Bowtie2 version 2.3.2^44^ with parameters -D 20 -R 3 -N 1 -L 20 -i S,1,0.50. If both forward and reverse reads of a paired-end sequence mapped to the same MGE, then it was counted as one; if they both mapped to different MGEs, they were counted toward those specific MGEs.^43^ MGE counts in each sample were aggregated based on MGE type.

### Taxonomic profiling

Whole shotgun metagenomic sequences were used for species-level taxonomic profiling using Metaphlan3 software^31^ that uses a library of clade-specific markers to provide pan-microbial (bacterial, archaeal, viral, and eukaryotic) profiling. Taxonomic abundance profiles were obtained for each sample from each cohort. Data for species-level compositional profiles were log-transformed to create heat maps to show the influence of antibiotic use over time. Hierarchical clustering of the samples was performed to identify samples that had similar species-level bacterial profiles, and a dendrogram was created using the ward.D2 method. For better interpretation, the order of the listed bacterial species in each heatmap was standardized from a roster that captured all species identified across all antibiotics. Species diversity was measured using sample alpha, within-sample diversity accounting for the different species observed, and beta diversity, measuring variation between all samples in a treatment group based on observed species. Alpha diversity was measured using the Shannon index, an index that accounts for both the number of species in a habitat and the relative abundance of those species. Beta diversity was measured using principal coordinate analysis (PCoA) of the samples.

### Statistical analysis

R packages metagenomeSeq,^45^ edgeR,^46^ ggplot2,^47^ and vegan^36^ were used for analysis. edgeR^46^ was used to determine differentially expressed populations of species from Metaphlan2. Adjusted population counts were determined by dividing the abundance by bacterial gene counts. Populations were assumed to be differentially expressed if the adjusted *p*-values were < .05. The vegan package was used to calculate the alpha and beta diversity of species populations. Diversity measurements were plotted using ggplot2 R package. The fitZig function in metagenomeSeq was used to determine differentially expressed MGEs and ARGs. ARGs and MGEs were removed from the analysis for <10 counts summed across all samples in a treatment group, and ARGs and MGEs were considered differentially expressed if the FDR was < .05. ARGs and MGEs integrases and transposons were summed to show differences in relative abundance between treatment time and controls. Results were expressed as log fold-change (logFC) between the control and treatment groups. ARGs were graphed if any of the timepoints were differentially expressed from the control, and the relative abundance of the ARG at any time point was greater than the relative abundance of ARG at the control point and sorted by the relative abundance of the genes after 72 h of treatment. The low-dose monotherapy treatments had many more ARGs that fit the criteria stated above; therefore, so to best represent the changes shown, the ARGs graphed for the low-dose monotherapy treatments showed the three genes with the greatest logFC after 72 h compared to the control samples. PCoA figures were created for the ARG and MGE profiles for each sample.

## Results

### Taxonomic profiling

Cohorts for a combination of ampicillin and fosfomycin, a combination of ciprofloxacin and fosfomycin, and an ampicillin monotherapy cohort all at lower concentrations were tested in mice, but an insufficient amount of DNA was collected for sequencing; therefore, these groups are absent in results (Table 1).

Heatmaps were created for the log-transformed abundance of the bacterial species-level taxonomic compositional profiles. Supplementary Figure S1 shows the abundance of all control samples from all cohorts tested, grouping the naïve and excipient treatment control groups together. Taxonomic profiles for all control samples were highly similar between all cohorts, with samples from the Amp_Cipro_High cohort being the exception, where the excipient treatment control group showed abundance that was lower and less diverse for all bacterial species. The PCoA plot (Supplementary Figure S2) based on Bray–Curtis dissimilarity of ARG abundances of all samples from all cohorts demonstrated that both naïve and excipient treatment control group samples were sufficiently similar to congregate in a single control group. Therefore, during downstream analysis, the naïve and excipient treatment control groups within each cohort were combined into a single group of control samples.

**Figure 1.**
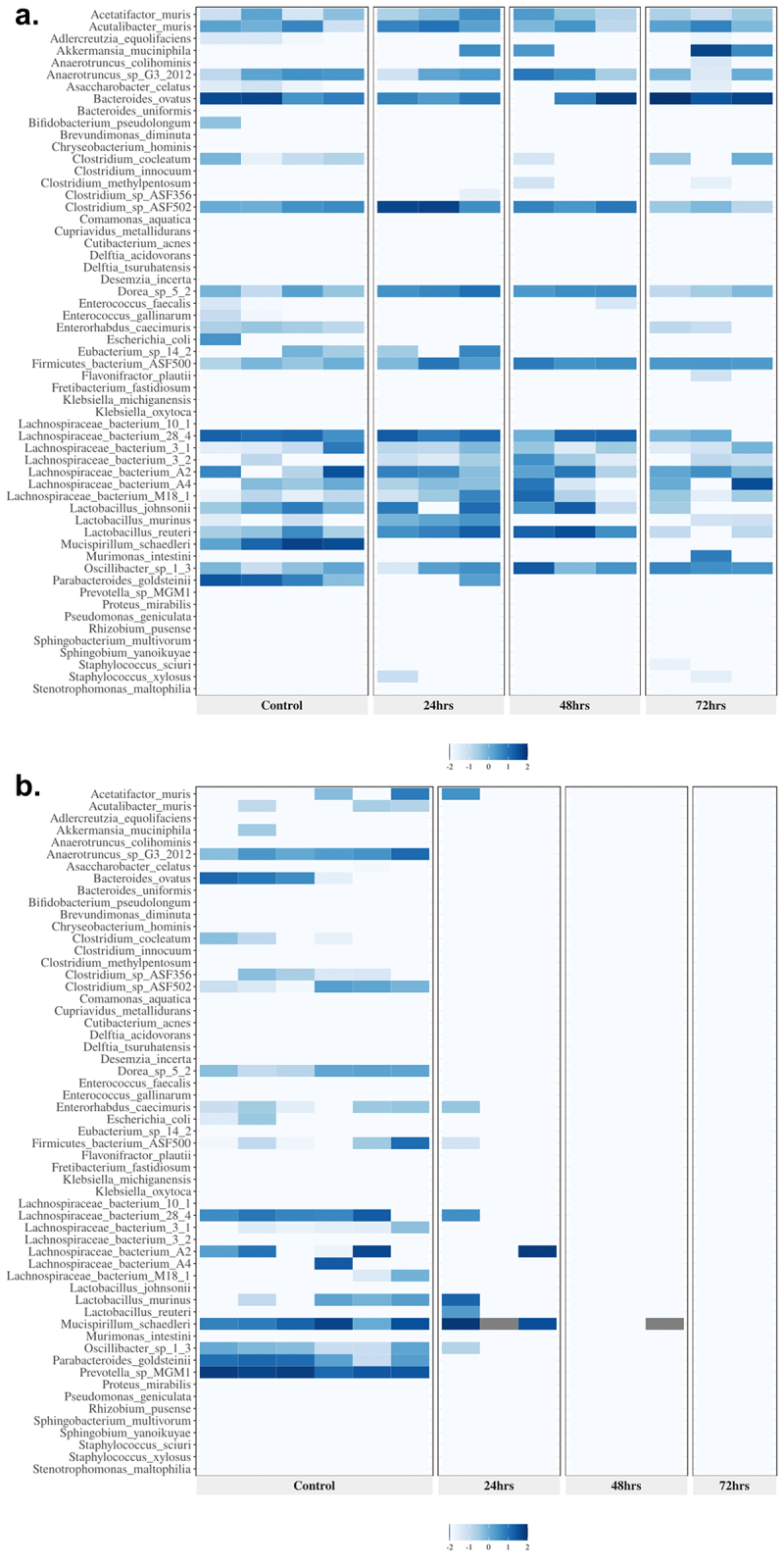
Heatmap presentation of antibiotic modulation of the log-transformed relative abundance of microbial species in the gut by combination and monotherapy after 24, 48, and 72 h of treatment; the two heatmaps shown are representative of (a) those seen in both low-dose monotherapies with sufficient DNA for analysis and (b) those seen in all combination therapy treatments: (a) low-dose ciprofloxacin and (b) triple combination low-dose ampicillin, ciprofloxacin, fosfomycin (also representative of all dual combination findings). These heatmaps present the species in the same order across each heatmap to allow comparisons. Color intensity indicates the relative abundance data (darker the color the greater the abundance) after log transformation.

Figure 1a represents the hierarchical clustering of samples based on taxonomy, shown is the ciprofloxacin low-dose monotherapy cohort that is typical of the clustering seen with the low-dose monotherapy groups, and Figure 1b shows the results for the triple combination high-dose Amp_Cipro_Fosfo_High group, typical of results from all combination treatments. Hierarchical clustering of all treatment groups is shown in Supplemental Figure S3. Generally, the control samples clustered together to the left side of the figures, with the treatment samples clustering to the right side of the figures. The previously mentioned Amp_Cipro_High cohort is the exception, where the excipient treatment control samples clustered more closely with the 48- and 72-h treatment samples, while the 24 h samples clustered with the naïve control samples. All treatment samples showed remarkably lower population abundances than the control samples, with less heterogeneity of species.

**Figure 2.**
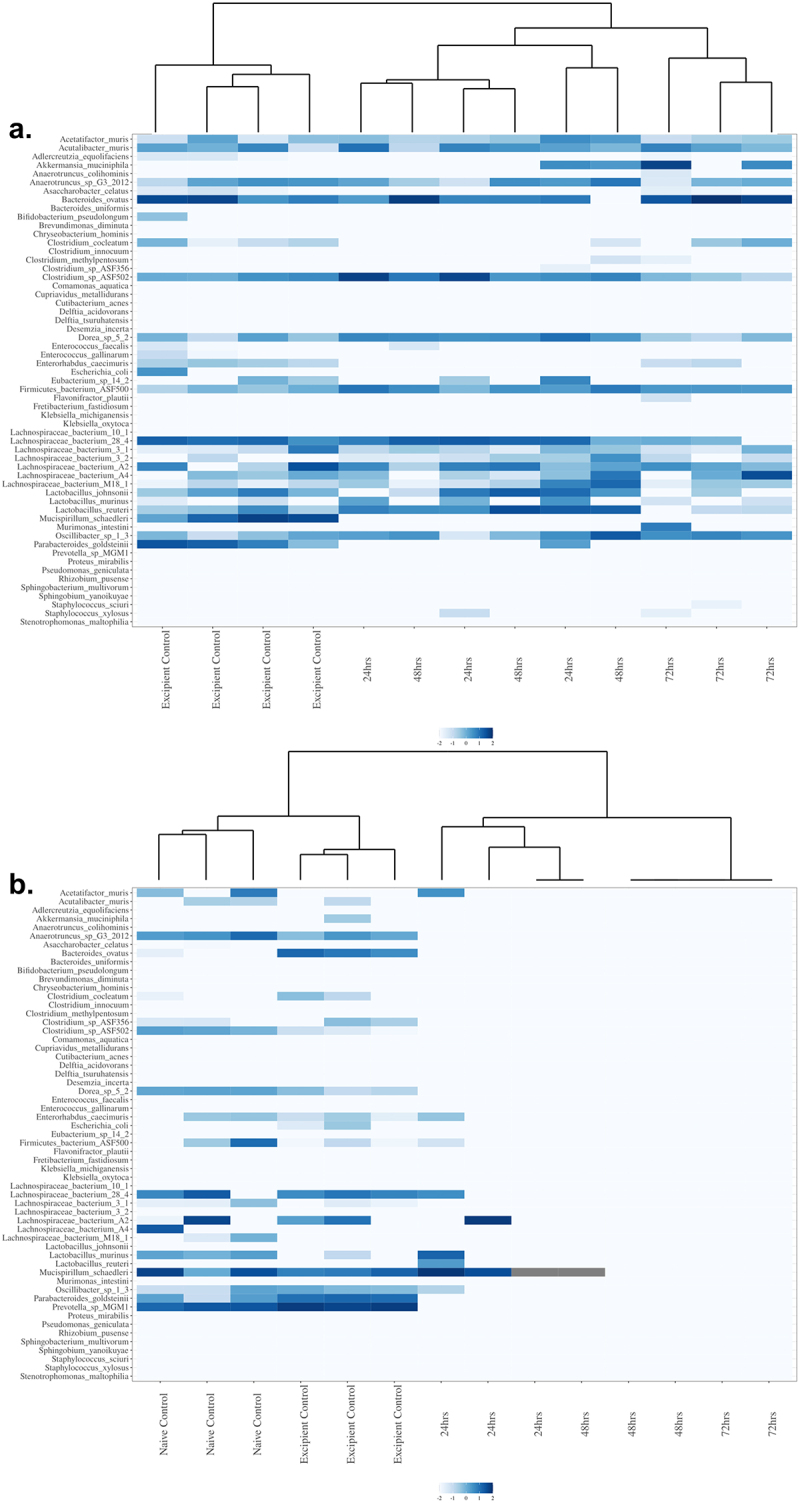
Heatmap with dendrogram demonstrating log-transformed relative abundance and clustering of bacterial species in the mouse gut after 24, 48, and 72 h of treatment. (a) Low-dose ciprofloxacin. (b) Triple combination high-dose ampicillin, ciprofloxacin, fosfomycin. Note the clustering together of control versus the clustering together of treated mice. Species were ordered in each graph to facilitate visualization of clustering. Color indicates the relative abundance data after log transformation.

Figure 2 shows representative heatmaps for bacterial taxonomic abundance displayed as treatment groups versus control samples; the differential expression of abundances for all cohorts is shown in Supplementary Table S2. Figure 2a shows the hierarchical clustering of the samples based on taxonomy. Shown is the ciprofloxacin low-dose monotherapy that is typical of the clustering seen with the low-dose monotherapy groups in the study and Figure 2b shows results from the triple combination high-dose Amp_Cipro_Fosfo_High group, typical of all combination treatments. All remaining heatmaps are shown can be found in Supplementary Figure S4. Controls generally showed similar abundance profiles for all cohorts, including high abundance of *Lachnospiraceae* spp., *Lactobacillus* spp. *Mucispirillum* schaerdleri, Parabacteriodes *goldstenii*, and *Prevotella* sp. *MGM1*. Low-dose monotherapy treatments of ciprofloxacin and fosfomycin both had few changes in taxonomic profiles following treatment. After treatment, the dual combination high-dose groups (Supplementary Figure S4) showed similar results to the triple combination group with few species surviving compared to controls, but those that remained were highly abundant. Several conditionally pathogenic organisms that have previously been shown the potential to be MDR survived dual combination treatment, these include *Enterococcus faecalis, Delftia acidovorans, Delftia tsuruhatensis*, and *Stenotrophomonas maltophilia*. Low-dose triple and dual combination groups (Supplementary Figure S4) showed similar taxonomic profiles after treatment, with fewer species than the control groups, but more than the high-dose combination treatments, and again included the conditionally pathogenic organisms listed above. Species populations showed immediate and dramatic changes after treatment with the triple combination high-dose treatment group (Figure 2b), and after 48 h of treatment there was only abundance of one species, *M. schaerdleri*, a common commensal organism in mice, and no abundance of any species after 72 h of treatment.

**Figure 3.**
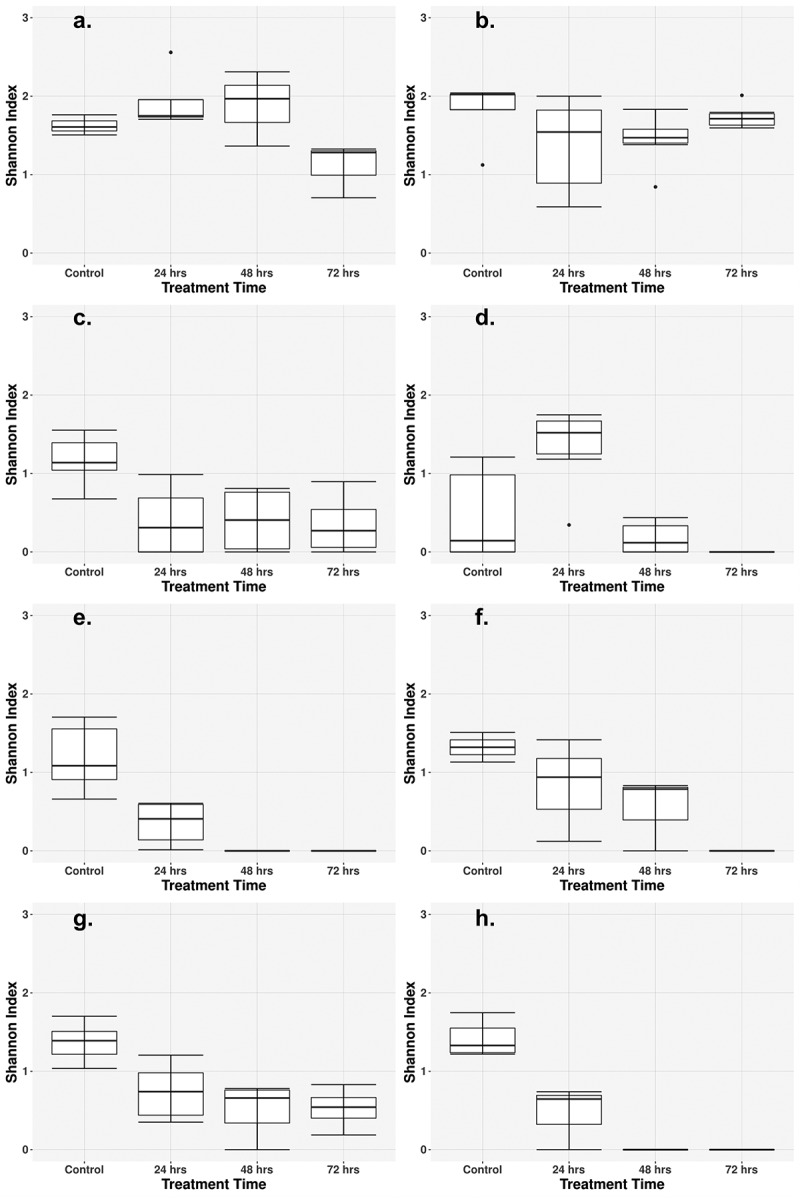
Longitudinal change of microbiome diversity after antibiotic treatment. Time-dependent change of the microbiome diversity, calculated as the Shannon diversity index based on bacterial abundances from Metaphlan3. (a) Low-dose ciprofloxacin. (b) Low-dose fosfomycin. (c) Low-dose combination ampicillin, ciprofloxacin. (d) High-dose combination ampicillin, ciprofloxacin. (e) High-dose combination ampicillin, fosfomycin. (f) High-dose combination ciprofloxacin, fosfomycin. (g) Triple combination low-dose ampicillin, ciprofloxacin, fosfomycin. (h) Triple combination high-dose ampicillin, ciprofloxacin, fosfomycin. Combination treatment at a higher dose caused a greater decrease in diversity, whereas low-dose treatments caused a less detectable decline after 72 h. Low-dose monotherapy treatments saw little to no change in diversity.

Generally, alpha diversity, within sample diversity, decreased for all cohorts after the 24-h treatment time and continued to decrease with successive treatment (Figure 3a–h). This trend was less pronounced with the single-class monotherapy antibiotic treatments and increasingly more pronounced with the higher dose combination therapies, and especially in the high-dose triple combination treatment. The Cipro_Low and Fosfo_Low cohorts differed from the combination treatment cohorts, as very little reduction was seen in alpha diversity after the 72-h treatment (Figure 3g–h). Most combination therapy groups showed a progressive decrease in diversity at each sampling time, with a less pronounced effect observed in the two low-dose combination therapy groups. Amp_Cipro_High did not follow the same trend as the alpha diversity increased after the 24-h treatment time, but then decreased after the 48-h treatment, and decreased to 0 after the 72-h treatment (Figure 3d). The Amp_Cipro_Fosfo_High cohort showed a large decrease after the first 24-h treatment followed by a diversity measurement of 0 for 48 and 72 h.

**Figure 4.**
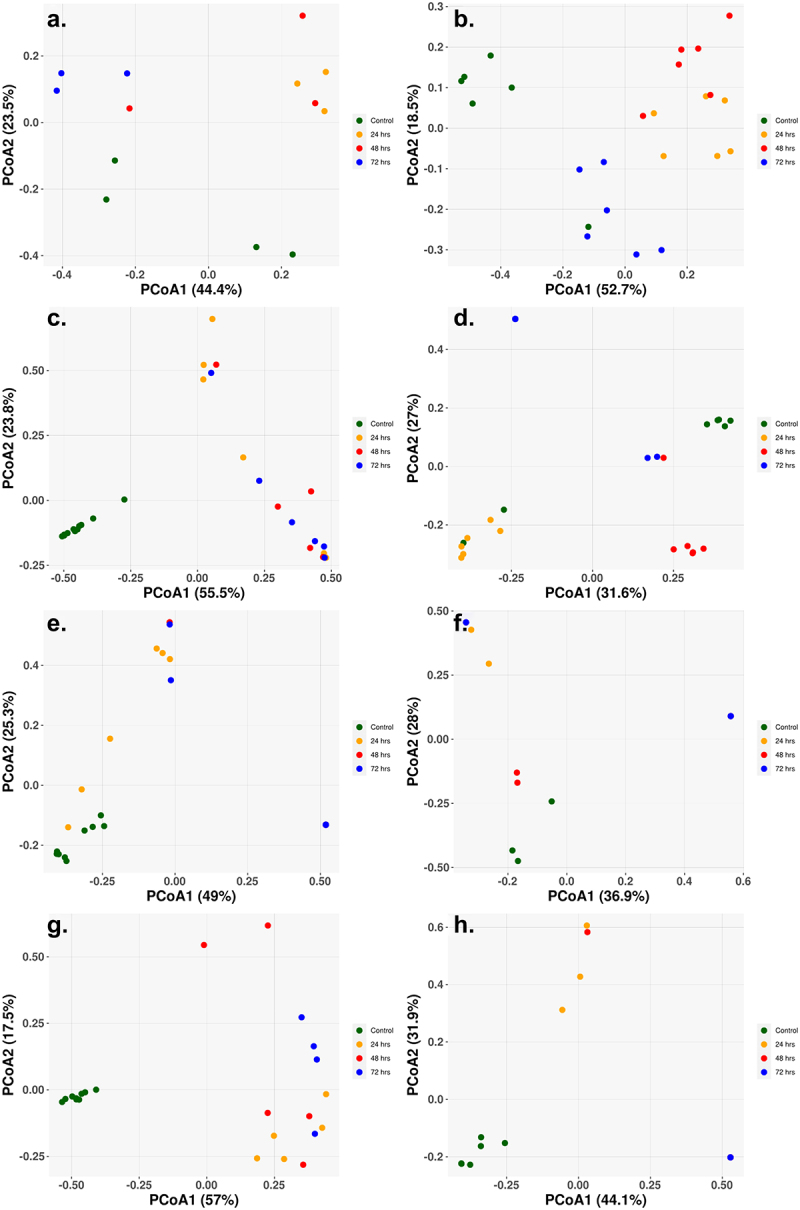
Principal coordinate of analysis (PCoA) reveals the time-dependent shift of metagenome profiles after oral treatment. (a) Low-dose ciprofloxacin. (b) Low-dose fosfomycin. (c) Low-dose combination ampicillin, ciprofloxacin. (d) High-dose combination ampicillin, ciprofloxacin. (e) High-dose combination ampicillin, fosfomycin. (f) High-dose combination ciprofloxacin, fosfomycin. (g) Triple combination low-dose ampicillin, ciprofloxacin, fosfomycin. (h) triple combination high-dose ampicillin, ciprofloxacin, fosfomycin. For each antibiotic cohort, the bacteria genera identified from each sample (solid dots) were subject to PCoA and the first and second principal coordinates are shown as *X-* and *Y*-axis, respectively. Within all three cohorts, there was a general trend in the way samples grouped together. Control samples grouped together and away from treated samples (24, 48, 72 h) indicating a change in genus profiles after treatment. The only exception being the combination of ampicillin and ciprofloxacin at a high dose where two of the control samples grouped with the 24 h and 72 h samples.

**Figure 5.**
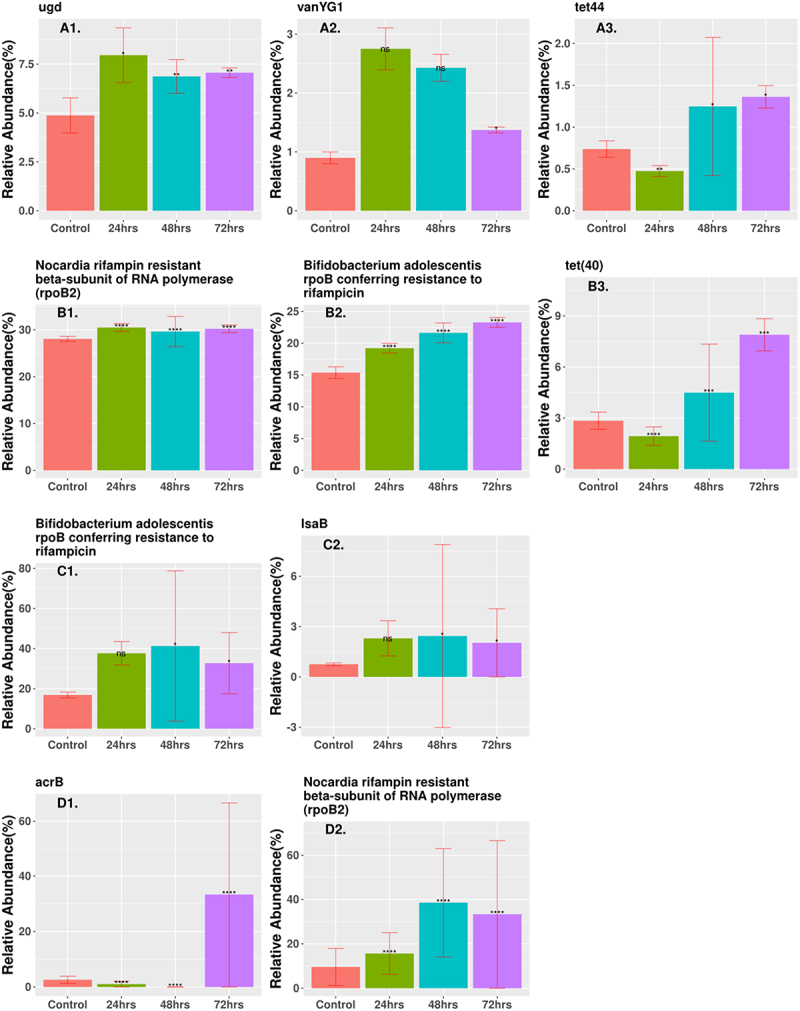
Relative abundance of enriched ARGs (CARD database) that are statistically significant and differentially represented after the final 72-h time point in samples treated with antibiotics. Genes were ordered based on relative abundance of the gene at the 72 h time point and the three highest abundance genes were shown. (*A1 ugd*, *A2 vanYG1*, *A3 tet44*) low-dose ciprofloxacin. (B1 rpoB2, B2 rpoB, B3 tet40) low-dose fosfomycin. (C1 rpoB, C2 lsaB) high-dose combination ampicillin, fosfomycin. (D1 acrB, D2 rpoB2) high-dose combination ciprofloxacin, fosfomycin. The height of each bar corresponds to the average relative abundance of the ARG for that specific timepoint. The relative abundance was calculated as the percentage of reads mapped to each ARG within the sample. Statistically significant change of relative abundance was defined as those pairwise comparisons between control and any time points with an FDR ≤ 0.05 (*FDR ≤0.05, ** FDR ≤ 0.01, *** FDR ≤ 0.001, **** FDR ≤ 0.0001). The triple combination high-dose ampicillin, ciprofloxacin, fosfomycin, triple combination low-dose ampicillin, ciprofloxacin, fosfomycin, high-dose combination ampicillin, ciprofloxacin, low-dose combination ampicillin, ciprofloxacin, and high-dose combination ciprofloxacin, fosfomycin had no genes that were statistically significant and differentially represented at the 72-h time point. Figure shows only a representative subset of ARGs that were significant at the 72-h time point.

The beta diversity, the between sample diversity, for each of the cohorts showed a clear separation between the control and sample treatments, with little separation distinction between the different treatment times (Figure 4a–h). The single class low-dosage antibiotic treatments showed similar results with clear separation of the control and treatment samples, despite only slight changes in the abundance populations (Figure 4g–h). The Amp_Cipro_High cohort differs from the other cohorts as the control samples are not clustered closely together, with two control samples on the far left and the remaining on the far right (Figure 5c). Figure 4h for the high-dose triple combination therapy shows the shift over time in the gut microbiome composition, with all control samples clustered to the bottom left of the figure, and as treatment begins and composition shifts, the samples cluster toward the top of the figure, and finally, as samples have little to no abundance after 72-h treatment, they cluster to the bottom right.

### Resistome profiling

ARG profiles are displayed in two different manners by relative abundance and PCoA of samples. Figures 5a–d show the relative abundance of any gene that was enriched and differentially expressed from the control at the 72 h time point. All other genes that displayed a significant differential expression and enrichment at any time point are listed in Table 2. Most genes showed a decrease in expression based on logFC for the combination therapy cohorts, while low-dose monotherapy cohorts showed several genes with an increase in gene count (Supplementary Table S3). The two low-dose monotherapy treatments had several genes that showed increases in relative abundance and were significantly expressed, with the three highest abundance genes shown in Figure 5(a,b). Cipro_Low showed immediate enrichment for *ugd* and *vanYG1*, while *tet44* decreased in abundance after 24 h but then showed a steady increase. Each differentially expressed gene in the Fosfo_Low cohort, *rpoB2*, *rpoB*, and *tet40*, showed steadily increased abundance at each treatment time. The Amp_Fosfo_High cohort showed similar increasing trends for *lsaB and rpoB* after 72 h (Figure 5c). Two genes in the Cipro_Fosfo_High cohort, *acrB* and *rpoB2*, also showed sharp increases in relative abundance after 72 h of treatment (Figure 5d). The high and low dual combination cohorts of Ampicillin and Ciprofloxacin, as well as the high and low triple combinations did not show any ARGs with increases in relative abundance and differential expression after 72-h treatment.

**Table 2.** All statistically significant ARGs that are enriched after oral treatment at any time point. (A) Low-dose ciprofloxacin. (B) Low-dose fosfomycin. (C) Low-dose combination ampicillin, ciprofloxacin. (D) High-dose combination ampicillin, ciprofloxacin. (E) High-dose combination ampicillin, fosfomycin. (F) High-dose combination ciprofloxacin, fosfomycin. (G) Triple combination high-dose ampicillin, ciprofloxacin, fosfomycin. (H) Triple combination low-dose ampicillin, ciprofloxacin, fosfomycin.

ARG	Increase/Decrease		
	24 h	48 h	72 h
**A. Ciprofloxacin Low Dose**			
RanA	-	-	+
amrB		+	+
mexY	+	+	+
tet44	-	+	+
ugd	+	+	+
vanG	+	-	-
vanRA	+	+	-
vanRD	+	-	-
vanRG	+	+	-
vanTG	-	+	+
vanYG1	+	+	+
**B. Fosfomycin Low Dose**			
AAC(6’)-Iih	-	-	+
ANT(6)-Ib	-	-	+
Bifidobacterium adolescentis rpoB conferring resistance to rifampicin	+	+	+
LlmA 23S ribosomal RNA methyltransferase	+	+	+
Nocardia rifampin-resistant beta-subunit of RNA polymerase (rpoB2)	+	+	+
RanA	+	+	+
RanB	+	+	+
TaeA	+	-	-
Txr	+	+	+
bcrA	+	+	-
cepA beta-lactamase	+	+	+
cmeB	+	+	+
cprR	+	+	+
efrB	+	+	+
macB	+	+	+
mdsB	+	+	+
mel	+	+	+
mexW	+	+	+
mupA	+	+	-
mupB	+	+	+
optrA	+	+	+
smeR	+	+	+
tet(40)	-	+	+
tet(W/N/W)	-	-	+
tet37	+	+	+
tetW	-	-	+
vanRA	-	-	+
vanRL	+	+	+
vanVB	-	-	+
vanXD	-	-	+
vanXYC	+	+	-
vanYB	+	+	+
vanYD	-	-	+
vanYG1	+	+	+
**C. Ampicillin; Ciprofloxacin Low Dose**			
efrA	+	+	+
Tet (40)	+	-	-
vanG	+	-	-
**D. Ampicillin; Ciprofloxacin High Dose**			
tetW	+	+	-
vanSN	+	-	-
**E. Ampicillin; Fosfomycin High Dose**			
Bifidobacterium adolescentis rpoB conferring resistance to rifampicin	+	+	+
LlmA 23S ribosomal RNA methyltransferase	-	+	-
acrD	+	-	-
lsaB	+	+	+
mupB	+	+	-
**F. Ciprofloxacin; Fosfomycin High Dose**			
Bifidobacterium adolescentis rpoB conferring resistance to rifampicin	+	-	-
*Escherichia coli* ampC	-	+	-
*Escherichia coli* ampH	-	+	-
*Klebsiella pneumoniae* KpnH	+	-	-
Nocardia rifampin-resistant beta-subunit of RNA polymerase (rpoB2)	+	+	+
OmpA	+	-	-
acrB	-	-	+
acrS	-	+	-
arnA	+	-	-
bacA	+	-	-
cpxA	+	-	-
efrA	+	-	-
efrB	-	+	-
emeA	-	+	-
emrK	+	+	-
emrR	+	-	-
evgA	+	-	-
macB	-	+	-
mdtB	+	-	-
msbA	+	-	-
rosB	+	+	-
tetO	+	-	-
tetS	-	+	-
ugd	-	+	-
vanSC	+	-	-
vanTC	-	+	-
vanYD	-	+	-
**G. Ampicillin; Ciprofloxacin; Fosfomycin Low Dose**			
macB	-	+	-
**H. Ampicillin; Ciprofloxacin; Fosfomycin High Dose**			
vanRD	+	-	-

**Figure 6.**
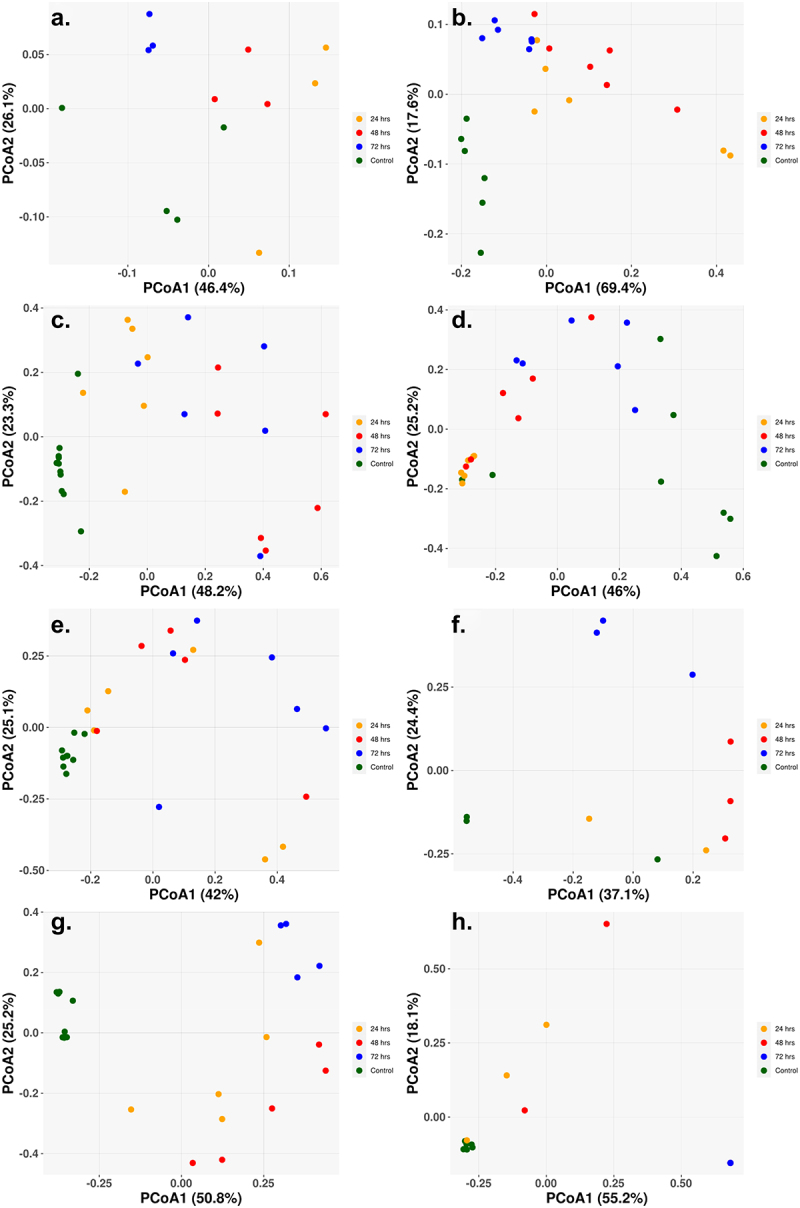
Principal coordinate of analysis (PCoA) based on Bray–Curtis dissimilarity of ARG abundances between samples reveals the time dependent shift of ARG profiles after oral treatment. (a) Low-dose ciprofloxacin. (b) Low-dose fosfomycin. (c) Low-dose combination ampicillin, ciprofloxacin. (d) High-dose combination ampicillin, ciprofloxacin. (e) High-dose combination ampicillin, fosfomycin. (f) High-dose combination ciprofloxacin, fosfomycin. (g) Triple combination low-dose ampicillin, ciprofloxacin, fosfomycin. (h) Triple combination high-dose ampicillin, ciprofloxacin, fosfomycin. For each antibiotic cohort, the first and second principal components are shown on *X*- and *Y*-axis, respectively. For all three cohorts, there was a general trend that control samples grouped together along either the *X-* and/or *Y*-axis, indicating control samples had similar profiles of ARG.

PCoA figures were created for the ARG profiles of each cohort, explaining the differences between the ARG profiles of each sample within the cohort Figure 6a–h. Generally, for all cohorts, there was clear separation in the PCoA between the control samples and the treatment samples, as well as separation between the treatment times. This was especially surprising for the low-dose monotherapy treatments, as they still contained a high abundance of ARGs with seemingly little change in bacterial composition profiles.

**Figure 7.**
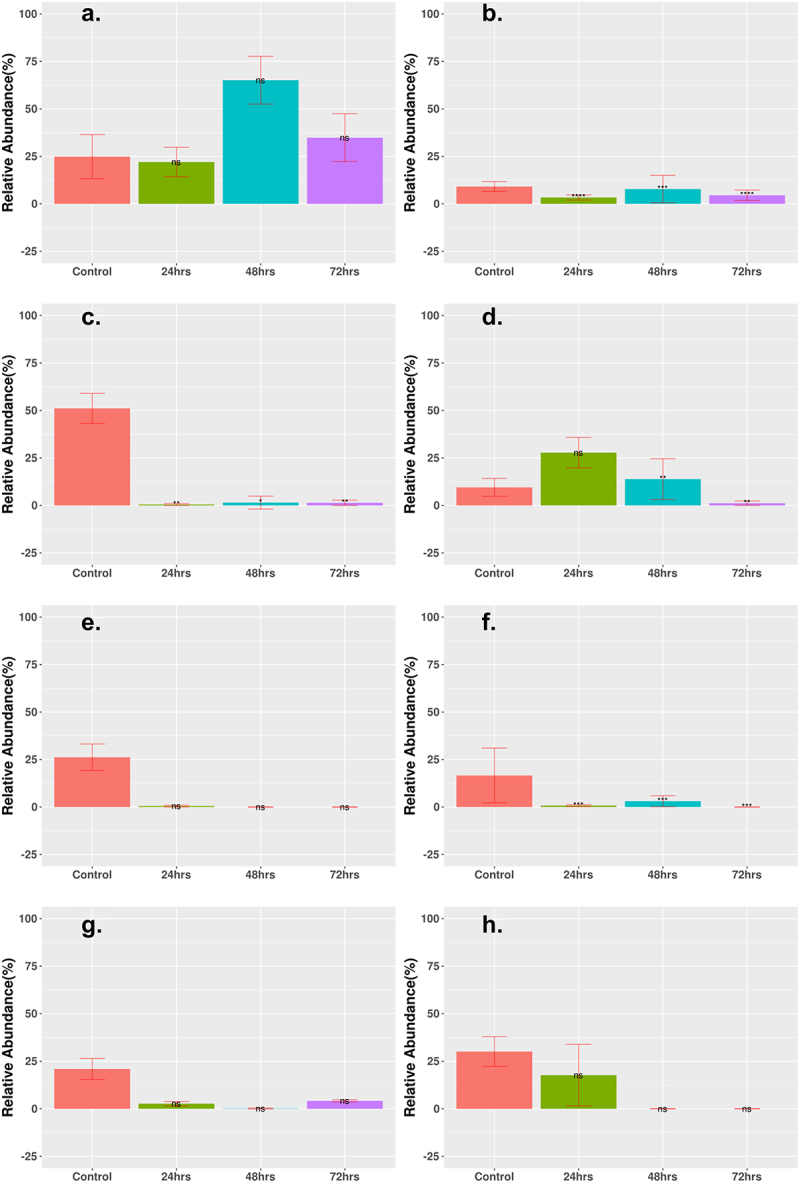
Statistically significant change in relative abundance of MGE integrase between control and treatment groups. Relative abundance of MGE integrase that had statistically significant change after treatment as shown. (a) Low-dose ciprofloxacin. (b) Low-dose fosfomycin. (c) Low-dose combination ampicillin, ciprofloxacin. (d) High-dose combination ampicillin, ciprofloxacin. (e) High-dose combination ampicillin, fosfomycin. (f) High-dose combination ciprofloxacin, fosfomycin. (g) Triple combination low-dose ampicillin, ciprofloxacin, fosfomycin. (h) Triple combination high-dose ampicillin, ciprofloxacin, fosfomycin. The height of each bar corresponds to the average relative abundance of integrase for that specific timepoint. The relative abundance was calculated as the percentage of reads mapped to each MGE within the sample. Statistically significant change of relative abundance was defined as those pairwise comparisons between control and 24, 48, and 72-h time points with an FDR ≤ 0.05 (*FDR ≤ 0.05, **FDR ≤ 0.01, ***FDR ≤ 0.001, ****FDR ≤ 0.0001, ns FDR > 0.05).

**Figure 8.**
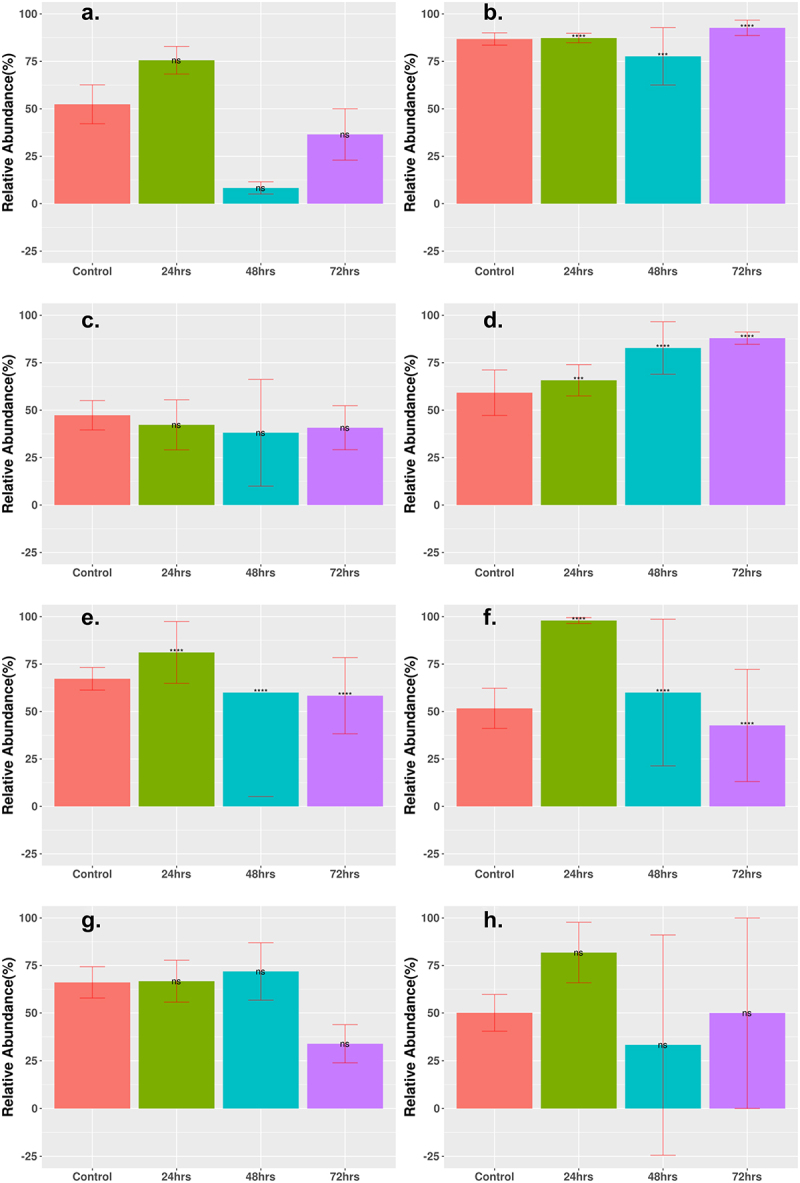
Statistically significant change in relative abundance of MGE transposase between control and treatment groups. Relative abundance of MGE integrase that had statistically significant change are shown. (a) Low-dose ciprofloxacin. (b) Low-dose fosfomycin. (c) Low-dose combination ampicillin, ciprofloxacin. (d) High-dose combination ampicillin, ciprofloxacin. (e) High-dose combination ampicillin, fosfomycin. (f) High-dose combination ciprofloxacin, fosfomycin. (g) Triple combination low-dose ampicillin, ciprofloxacin, fosfomycin. (h) Triple combination high-dose ampicillin, ciprofloxacin, fosfomycin. The height of each bar corresponds to the average relative abundance of integrase for that specific timepoint. The relative abundance was calculated as the percentage of reads mapped to each MGE within the sample. Statistically significant change of relative abundance was defined as those pairwise comparisons between control and 24, 48, and 72 h time points with (*FDR ≤ 0.05, **FDR ≤ 0.01, ***FDR ≤ 0.001, ****FDR ≤ 0.0001, ns FDR > 0.05).

MGE counts were grouped according to the abundance of two major categories: integrases and transposases. Generally, for all cohorts, the abundance of integrases decreased after 72 h of treatment (Figure 7a–h), whereas the abundance of transposases either increased slightly or remained the same after treatment (Figure 8a–h). The logFC for most time points showed a decrease (Supplementary Table S4). All cohorts, aside from the Cipro_Low cohort, showed a decrease of integrases to <5% relative abundance of total MGEs after 72-h treatment time. The results for transposase abundance were less consistent, as the Fosfo_Low and Amp_Cirpo_High cohorts showed significant increases in relative abundance after the 72-h treatment. The abundance of transposases decreased or remained the same for all the remaining cohorts after the 72-h treatment time with a final relative abundance ranging from ~30% to 60%. The final relative abundance for the Amp_Cipro_Fosfo_High cohort remained the same as that of the control cohort and did not show a significant difference.

## Discussion

Combination therapy, which involves the use of multiple classes of antibiotics, has been explored as a strategy to prevent the emergence of resistant bacteria when treating cystitis and other common community-acquired infections. Clinical concerns that have been raised about combination therapy include increased toxicity compared with single agents,^37,48^ an increased risk for *Clostridioides difficile* infection, and the possible emergence of MDR bacteria.^49,50^ While combinations of antibiotics have been used to treat MDR bacterial infections in a clinical setting,^51^ the off-target effect on the emergence of ARGs and the overall effect on the composition of the gut bacterial profile are not well understood.^52^ In addition to looking at different combinations and different doses of combinations, this study also examined the effect of low-dose monotherapy that might represent outcomes with inadequate antibiotic exposures.

In the present analysis, all treatments decreased the alpha and beta diversity of the microbiome. Assuming that antibiotics will kill bacteria, this narrowing of diversity would be expected. Our results showed that combinations of antibiotics further reduced the diversity of species in gut flora compared to monotherapy. In a broad comparison to our original research from Xu et al.,^1^ which tested the change in mouse gut microbiome and resistome using high-dose single-class monotherapy, combination therapy reduced the total measurable gut bacterial population. The overall species diversity and abundance were much lower after 72 h of treatment in the high-dose dual combination treatments compared to the single class treatments, while the low-dose triple and dual combination therapy cohorts had results similar to dual high-dose triple combination cohort. After treatment, the low-dose monotherapy cohorts showed a greater final species abundance and diversity compared to the high-dose monotherapy cohorts in the original research.^1^

Low-dose monotherapy treatments showed an increase in the abundance of ARGs compared to control and high-dose monotherapy treatments. There were even fewer ARGs that significantly increased in abundance after 72 h of treatment for all combination therapy groups, including the low-dose combinations, and the results for MGE abundance were similar for all treatment cohorts, including those in the original study, with a sharp decrease in the abundance of integrases after treatment, while transposase abundance was generally lower in combination therapy compared to high-dose monotherapy in the original study. The high-dose triple combination therapy showed the greatest reduction in species abundance and diversity and in ARG abundance and diversity compared across all cohorts from our current study and the study by Xu et al.^1^

In the present study, four organisms were found in the final microbiome profiles that were previously shown to be potentially MDR and have conditionally caused infections in prior clinical settings. These organisms, *E. faecalis*,^53^ *D. acidovorans*,^54^ *D. tsuruhatensis*, and *S. maltophila* ^55^ were separately found in the final taxonomic abundance profiles of the ampicillin, ciprofloxacin high- and low-dose combination, the ampicillin, fosfomycin high-dose combination, and the ampicillin, ciprofloxacin, fosfomycin low-dose triple combination therapy. These organisms were not uniformly found, and it is unclear what their relevance would be in a clinical setting. There was no abundance of any conditionally pathogenic organisms found after lower dose monotherapy treatments, possibly due to competitive exclusion because there were only minor shifts in taxonomic profiles.^56^ Despite the minor changes in microbial abundance, samples were clearly separated based on beta diversity (Figure 4), indicating that there is an effect of low doses of antibiotics on the microbiome. Recently published metagenomic studies on both murine (Leclercq)^57^ and avian (Ward)^58^ intestinal tracts have shown a detectable shift in microbial populations when low doses of antibiotics were administered.^57,58^ The triple combination high-dose therapy of ampicillin, ciprofloxacin, and fosfomycin showed no abundance of conditionally pathogenic organisms after treatment, an encouraging sign that there will be no unintended off target effects for the use of combination therapy in the treatment of infections.

Low-dose monotherapy cohorts showed a greater increase in ARG abundance after treatment than combination therapies, suggesting that low-dose monotherapy may drive the emergence of antimicrobial resistance in the host with potential spread to the environment (Figure 5, Supp Table S3). After 72 h, the Cipro_Low cohort was enriched for *ugd* causing resistance in polymyxins, especially in enteric diseases,^59^ *vanYG1* conferring resistance to vancomycin,^60^ and *tet(44)* that is likely to confer resistance to tetracycline by ribosomal protection.^61^ The low-dose fosfomycin cohort was enriched for *rpoB2* that confers resistance to rifampicin,^62^ *rpoB* that confers resistance to rifampicin,^63^ and *tet40* a tetracycline efflux gene.^64^ The Amp_Fosfo_High cohort showed increases in *lsaB* that confers resistance to clindamycin and *rpoB* that confers resistance to rifampicin. The Cipro_Fosfo_High cohort showed increases in *rpoB2* that confers resistance to rifampicin, and *acrB* which encodes an efflux pump causing resistance in fluoroquinolones (class including cirpofloxacin) for both *Salmonella enterica* and *E. coli*. ^65,66^ Other studies have shown enrichment of several efflux pumps that are capable of conferring cross-resistance to multiple antibiotics.^67^ The Amp_Cipro_Fosfo_High cohort showed no increase in the abundance of any ARGs, most likely due to a combination of total reduction in all genetic information and suppression of ARGs from the combination of classes of antibiotics.

The final abundance of integrases and transposases was generally greater in the low-dose monotherapy treatments than in the combination therapy treatments. MGEs are an important part of how ARGs can spread inside the gut microbiome,^43,68^ and the sharp decrease in the abundance of MGEs may be one possible explanation for the lack of increase in ARGs over the course of treatment time. There was little change in the abundance of integrases or transposases in the low-dose monotherapy cohorts, and the lack of decline in the abundance of integrases and transposases may be a contributing factor to why the monotherapy treatments at a lower concentration saw a much greater increase in the abundance of several ARGs.

This study has several limitations. One limitation is the conversion from mouse to human equivalent dose (HED) in our study, specifically for fosfomycin treatment. A conversion range using typical mouse bodyweight (0.011–0.034 kg) with average global human bodyweight and North American average bodyweight (62–81 kg) gave a HED for fosfomycin of 2542–7857 mg/d in the high-dose group.^69,70^ The most common fosfomycin regimen is a single dose (3000 mg) to treat a single episode or a single dose every 3 d for multiple-dose treatment episodes.^71^ This study was also limited by the lack of a complete dataset due to the inability to recover adequate amounts of genomic material from all combinations. Specifically, inadequate amounts of DNA were available from low-dose dual cohorts. For the low-dose dual treatment groups, we included only the Amp_Cipro_Low group. Other dual combination groups may have shown different abilities to prevent the spread of ARGs and emergence of MDR bacteria. Why insufficient DNA was extracted is uncertain but could be related to variations in experimental procedures and/or the mouse cohorts. While the absence of this data prohibits some comparisons, but it does not negate the findings for cohorts for which sampling was complete. The results for the Amp_Cipro_High treatment group are inconsistent with all other findings, as it was the only cohort to show little to no changes in the gut taxonomic profile and resistome after 24 h, but then a sharp decline in bacterial populations and ARG and MGE abundance after 48 h. The authors have no explanation for this inconsistency. Additional experiments would be needed to determine whether this is an isolated occurrence or whether it would be observed in repeated experiments. In addition, our study was unable to detect novel ARGs and MGEs that may arise during treatment, as our methods were confined by ARGs and MGEs found in existing databases, CARD and MobileGeneticElementDatabase.

Low-dose monotherapy treatments led to increased abundance of several ARGs and MGEs, while also showing a minor, but detectable, change in gut microbiome profile. This suggests that inadequate exposure could promote emergence of resistance. High- and low-dose dual combination therapies and low-dose triple combination therapies were shown to effectively suppress the increase in abundance of ARGs seen in monotherapy but did allow for the emergence of some conditionally pathogenic organisms. Triple combination therapy treatment decreased the abundance of ARGs and prevented emergence of opportunistic pathogens. These results suggest that high-dose combination therapy for bacterial infections may suppress emergent resistance within the microbiome. Future studies should focus on the effects of repopulation of the gut microbiome after combination therapy, and on determining whether novel ARGs and MGEs arise after treatment.

## Supplementary Material

Supplemental Material

## References

[1] Xu L, Surathu A, Raplee I, Chockalingam A, Stewart S, Walker L, Sacks L, Patel V, Li Z, Rouse R, et al. The effect of antibiotics on the gut microbiome: a metagenomics analysis of microbial shift and gut antibiotic resistance in antibiotic treated mice. BMC Genom. 2020;21:263. doi:10.1186/s12864-020-6665-2. PMC7106814 32228448

[2] French GL. Clinical impact and relevance of antibiotic resistance. Adv Drug Deliv Rev. 2005;57:1514–22. doi:10.1016/j.addr.2005.04.005. 15978698

[3] van Duin D, Paterson DL. Multidrug-resistant bacteria in the community: trends and lessons learned. Infect Dis Clin North Am. 2016;30:377–390. doi:10.1016/j.idc.2016.02.004. 27208764 PMC5314345

[4] Antimicrobial Resistance C, Ikuta KS, Sharara F, Swetschinski L, Robles Aguilar G, Gray A, Han C, Bisignano C, Rao P, Wool E. Global burden of bacterial antimicrobial resistance in 2019: a systematic analysis. Lancet. 2022;399:629–655. doi:10.1016/S0140-6736(21)02724-0. 35065702 PMC8841637

[5] Raplee I, Walker L, Xu L, Surathu A, Chockalingam A, Stewart S, Han X, Rouse R, Li Z. Emergence of nosocomial associated opportunistic pathogens in the gut microbiome after antibiotic treatment. Antimicrob Resist Infect Control. 2021;10:36. doi:10.1186/s13756-021-00903-0. 33588951 PMC7885457

[6] Carattoli A. Plasmids and the spread of resistance. Int J Med Microbiol. 2013;303:298–304. doi:10.1016/j.ijmm.2013.02.001. 23499304

[7] Partridge SR, Kwong SM, Firth N, Jensen SO. Mobile genetic elements associated with antimicrobial resistance. Clin Microbiol Rev. 2018;31:31. doi:10.1128/CMR.00088-17. PMC6148190 30068738

[8] Broaders E, Gahan CG, Marchesi JR. Mobile genetic elements of the human gastrointestinal tract: potential for spread of antibiotic resistance genes. Gut Microbes. 2013;4:271–280. doi:10.4161/gmic.24627. 23651955 PMC3744512

[9] Stokes HW, Gillings MR. Gene flow, mobile genetic elements and the recruitment of antibiotic resistance genes into gram-negative pathogens. FEMS Microbiol Rev. 2011;35:790–819. doi:10.1111/j.1574-6976.2011.00273.x. 21517914

[10] Livermore DM. The need for new antibiotics. Clin Microbiol Infect. 2004;10(Suppl 4):1–9. doi:10.1111/j.1465-0691.2004.1004.x. 15522034

[11] Mouton JW. Combination therapy as a tool to prevent emergence of bacterial resistance. Infection. 1999;27 Suppl 2:S24–8. doi:10.1007/BF02561666. 10885823

[12] Paul M, Lador A, Grozinsky-Glasberg S, Leibovici L. Beta lactam antibiotic monotherapy versus beta lactam-aminoglycoside antibiotic combination therapy for sepsis. Cochrane Database Syst Rev. 2014;2014:CD003344. doi:10.1002/14651858.CD003344.pub3. 24395715 PMC6517128

[13] Safdar N, Handelsman J, Maki DG. Does combination antimicrobial therapy reduce mortality in gram-negative bacteraemia? A meta-analysis. Lancet Infect Dis. 2004;4:519–527. doi:10.1016/S1473-3099(04)01108-9. 15288826

[14] Fish DN, Piscitelli SC, Danziger LH. Development of resistance during antimicrobial therapy: a review of antibiotic classes and patient characteristics in 173 studies. Pharmacotherapy. 1995;15:279–291. 7667163

[15] Kosmidis J, Koratzanis G. Emergence of resistant bacterial strains during treatment of infections in the respiratory tract. Scand J Infect Dis Suppl. 1986;49:135–139. 3103208

[16] Bryan CS, Reynolds KL, Brenner ER. Analysis of 1,186 episodes of gram-negative bacteremia in non-university hospitals: the effects of antimicrobial therapy. Rev Infect Dis. 1983;5:629–638. doi:10.1093/clinids/5.4.629. 6353525

[17] Geerdes HF, Ziegler D, Lode H, Hund M, Loehr A, Fangmann W, Wagner J. Septicemia in 980 patients at a university hospital in Berlin: prospective studies during 4 selected years between 1979 and 1989. Clin Infect Dis. 1992;15:991–1002. doi:10.1093/clind/15.6.991. 1457672

[18] Hyle EP, Lipworth AD, Zaoutis TE, Nachamkin I, Bilker WB, Lautenbach E. Impact of inadequate initial antimicrobial therapy on mortality in infections due to extended-spectrum beta-lactamase-producing Enterobacteriaceae: variability by site of infection. Arch Intern Med. 2005;165:1375–1380. doi:10.1001/archinte.165.12.1375. 15983286

[19] Gionchetti P, Rizzello F, Venturi A, Ugolini F, Rossi M, Brigidi P, Johansson R, Ferrieri A, Poggioli G, Campieri M, et al. Antibiotic combination therapy in patients with chronic, treatment-resistant pouchitis. Aliment Pharmacol Ther. 1999;13:713–718. doi:10.1046/j.1365-2036.1999.00553.x. 10383499

[20] Acar JF. Antibiotic synergy and antagonism. Med Clin North Am. 2000;84:1391–1406. doi:10.1016/s0025-7125(05)70294-7. 11155849

[21] Torella JP, Chait R, Kishony R, Bourne PE. Optimal drug synergy in antimicrobial treatments. PLoS Comput Biol. 2010;6:e1000796. doi:10.1371/journal.pcbi.1000796. 20532210 PMC2880566

[22] Sullivan GJ, Delgado NN, Maharjan R, Cain AK. How antibiotics work together: molecular mechanisms behind combination therapy. Curr Opin Microbiol. 2020;57:31–40. doi:10.1016/j.mib.2020.05.012. 32619833

[23] Garimella N, Zere T, Hartman N, Gandhi A, Bekele A, Li X, Stone H, Sacks L, Weaver JL. Effect of drug combinations on the kinetics of antibiotic resistance emergence in Escherichia coli CFT073 using an in vitro hollow-fibre infection model. Int J Antimicrob Agents. 2020;55:105861. doi:10.1016/j.ijantimicag.2019.105861. 31838036

[24] Hugenholtz P, Tyson GW. Microbiology: metagenomics. Nature. 2008;455:481–483. doi:10.1038/455481a. 18818648

[25] Tringe SG, von Mering C, Kobayashi A, Salamov AA, Chen K, Chang HW, Podar M, Short JM, Mathur EJ, Detter JC, et al. Comparative metagenomics of microbial communities. Sci. 2005;308:554–557. doi:10.1126/science.1107851. 15845853

[26] Yadav S, Kapley A. Antibiotic resistance: global health crisis and metagenomics. Biotechnol Rep (Amst). 2021;29:e00604. doi:10.1016/j.btre.2021.e00604. 33732632 PMC7937537

[27] Sukhum KV, Diorio-Toth L, Dantas G. Genomic and metagenomic Approaches for Predictive Surveillance of Emerging pathogens and antibiotic resistance. Clin Pharmacol Ther. 2019;106:512–524. doi:10.1002/cpt.1535. 31172511 PMC6692204

[28] Schmieder R, Edwards R. Insights into antibiotic resistance through metagenomic approaches. Future Microbiol. 2012;7:73–89. doi:10.2217/fmb.11.135. 22191448

[29] Quince C, Walker AW, Simpson JT, Loman NJ, Segata N. Shotgun metagenomics, from sampling to analysis. Nat Biotechnol. 2017;35:833–844. doi:10.1038/nbt.3935. 28898207

[30] Jovel J, Patterson J, Wang W, Hotte N, O’Keefe S, Mitchel T, Perry T, Kao D, Mason AL, Madsen KL, et al. Characterization of the gut microbiome using 16S or shotgun metagenomics. Front Microbiol. 2016;7:459. doi:10.3389/fmicb.2016.00459. 27148170 PMC4837688

[31] Beghini F, McIver LJ, Blanco-Miguez A, Dubois L, Asnicar F, Maharjan S, Mailyan A, Manghi P, Scholz M, Thomas AM, et al. Integrating taxonomic, functional, and strain-level profiling of diverse microbial communities with bioBakery 3. Elife. 2021;10. doi:10.7554/eLife.65088. PMC8096432 33944776

[32] Bolyen E, Rideout JR, Dillon MR, Bokulich NA, Abnet CC, Al-Ghalith GA, Alexander H, Alm EJ, Arumugam M, Asnicar F, et al. Reproducible, interactive, scalable and extensible microbiome data science using QIIME 2. Nat Biotechnol. 2019;37:852–857. doi:10.1038/s41587-019-0209-9. 31341288 PMC7015180

[33] Mardis ER. DNA sequencing technologies: 2006–2016. Nat Protoc. 2017;12:213–218. doi:10.1038/nprot.2016.182. 28055035

[34] Chockalingam A, Stewart S, Xu L, Gandhi A, Matta MK, Patel V, Sacks L, Rouse R. Evaluation of immunocompetent urinary tract infected Balb/C mouse model for the study of antibiotic resistance development using Escherichia Coli CFT073 infection. Antibiot (Basel). 2019;8:170. doi:10.3390/antibiotics8040170. PMC6963567 31569374

[35] Peck MA, Sturk-Andreaggi K, Thomas JT, Oliver RS, Barritt-Ross S, Marshall C. Developmental validation of a Nextera XT mitogenome Illumina MiSeq sequencing method for high-quality samples. Forensic Sci Int Genet. 2018;34:25–36. doi:10.1016/j.fsigen.2018.01.004. 29413633

[36] Oksanen J, Simpson, G, Blanchet, F, Kindt R, Legendre P, Minchin, P, O’Hara R, Stevens M, Szoecs E, et al. The vegan package. Commun Eco Package. 2007;10:719.

[37] Roberts MC. Antibiotic toxicity, interactions and resistance development. Periodontol 2000. 2002;28:280–297. doi:10.1034/j.1600-0757.2002.280112.x. 12013346

[38] Li H, Durbin R. Fast and accurate short read alignment with Burrows-Wheeler transform. Bioinformatics. 2009;25:1754–1760. doi:10.1093/bioinformatics/btp324. 19451168 PMC2705234

[39] Li H, Handsaker B, Wysoker A, Fennell T, Ruan J, Homer N, Marth G, Abecasis G, Durbin R. The sequence alignment/map format and SAMtools. Bioinformatics. 2009;25:2078–2079. doi:10.1093/bioinformatics/btp352. 19505943 PMC2723002

[40] Broad Institute; Github Repository. Picard Toolkit. Broad Institute; 2019.

[41] Camacho C, Coulouris G, Avagyan V, Ma N, Papadopoulos J, Bealer K, Madden TL. BLAST+: architecture and applications. BMC Bioinform. 2009;10:421. doi:10.1186/1471-2105-10-421. PMC2803857 20003500

[42] McArthur AG, Waglechner N, Nizam F, Yan A, Azad MA, Baylay AJ, Bhullar K, Canova MJ, De Pascale G, Ejim L, et al. The comprehensive antibiotic resistance database. Antimicrob Agents Chemother. 2013;57:3348–3357. doi:10.1128/AAC.00419-13. 23650175 PMC3697360

[43] Parnanen K, Karkman A, Hultman J, Lyra C, Bengtsson-Palme J, Larsson DGJ, Rautava S, Isolauri E, Salminen S, Kumar H, et al. Maternal gut and breast milk microbiota affect infant gut antibiotic resistome and mobile genetic elements. Nat Commun. 2018;9:3891. doi:10.1038/s41467-018-06393-w. 30250208 PMC6155145

[44] Langmead B, Salzberg SL. Fast gapped-read alignment with bowtie 2. Nat Methods. 2012;9:357–359. doi:10.1038/nmeth.1923. 22388286 PMC3322381

[45] Paulson JN, Pop M, Bravo HC. metagenomeSeq: statistical analysis for sparse high-throughput sequencing. Bioconductor Package. 2013;1:191.

[46] Robinson MD, McCarthy DJ, Smyth GK. edgeR: a Bioconductor package for differential expression analysis of digital gene expression data. Bioinformatics. 2010;26:139–140. doi:10.1093/bioinformatics/btp616. 19910308 PMC2796818

[47] Wickham H, ggplo 2. Computational statistics. Wiley Interdisciplin Rev. 2011;3:180–185. doi:10.1002/wics.147.

[48] Paul M, Dickstein Y, Schlesinger A, Grozinsky-Glasberg S, Soares-Weiser K, Leibovici L. Beta-lactam versus beta-lactam-aminoglycoside combination therapy in cancer patients with neutropenia. Cochrane Database Syst Rev. 2013;2013:CD003038. doi:10.1002/14651858.CD003038.pub2. 23813455 PMC6457814

[49] Bliziotis IA, Samonis G, Vardakas KZ, Chrysanthopoulou S, Falagas ME. Effect of aminoglycoside and beta-lactam combination therapy versus beta-lactam monotherapy on the emergence of antimicrobial resistance: a meta-analysis of randomized, controlled trials. Clin Infect Dis. 2005;41:149–158. doi:10.1086/430912. 15983909

[50] Vestergaard M, Paulander W, Marvig RL, Clasen J, Jochumsen N, Molin S, Jelsbak L, Ingmer H, Folkesson A. Antibiotic combination therapy can select for broad-spectrum multidrug resistance in Pseudomonas aeruginosa. Int J Antimicrob Agents. 2016;47:48–55. doi:10.1016/j.ijantimicag.2015.09.014. 26597931

[51] Ahmed A, Azim A, Gurjar M, Baronia AK. Current concepts in combination antibiotic therapy for critically ill patients. Indian J Crit Care Med. 2014;18:310–314. doi:10.4103/0972-5229.132495. 24914260 PMC4047693

[52] Tamma PD, Cosgrove SE, Maragakis LL. Combination therapy for treatment of infections with gram-negative bacteria. Clin Microbiol Rev. 2012;25:450–470. doi:10.1128/CMR.05041-11. 22763634 PMC3416487

[53] Noskin GA, Peterson LR, Warren JR. Enterococcus faecium and Enterococcus faecalis bacteremia: acquisition and outcome. Clin Infect Dis. 1995;20:296–301. doi:10.1093/clinids/20.2.296. 7742433

[54] Bilgin H, Sarmis A, Tigen E, Soyletir G, Mulazimoglu L. Delftia acidovorans: a rare pathogen in immunocompetent and immunocompromised patients. Can J Infect Dis Med Microbiol. 2015;26:277–279. doi:10.1155/2015/973284. 26600818 PMC4644013

[55] Denton M, Kerr KG. Microbiological and clinical aspects of infection associated with Stenotrophomonas maltophilia. Clin Microbiol Rev. 1998;11:57–80. doi:10.1128/CMR.11.1.57. 9457429 PMC121376

[56] Nurmi E, Nuotio L, Schneitz C. The competitive exclusion concept: development and future. Int J Food Microbiol. 1992;15:237–240. doi:10.1016/0168-1605(92)90054-7. 1419528

[57] Leclercq S, Mian FM, Stanisz AM, Bindels LB, Cambier E, Ben-Amram H, Koren O, Forsythe P, Bienenstock J. Low-dose penicillin in early life induces long-term changes in murine gut microbiota, brain cytokines and behavior. Nat Commun. 2017;8:15062. doi:10.1038/ncomms15062. 28375200 PMC5382287

[58] Ward TL, Weber BP, Mendoza KM, Danzeisen JL, Llop K, Lang K, Clayton JB, Grace E, Brannon J, Radovic I, et al. Antibiotics and host-tailored probiotics similarly modulate effects on the developing avian microbiome, mycobiome, and host gene expression. mBio. 2019;10. doi:10.1128/mBio.02171-19. PMC6794479 31615957

[59] Lee H, Hsu FF, Turk J, Groisman EA. The PmrA-regulated pmrC gene mediates phosphoethanolamine modification of lipid a and polymyxin resistance in Salmonella enterica. J Bacteriol. 2004;186:4124–4133. doi:10.1128/JB.186.13.4124-4133.2004. 15205413 PMC421605

[60] Boyd DA, Du T, Hizon R, Kaplen B, Murphy T, Tyler S, Brown S, Jamieson F, Weiss K, Mulvey MR, et al. VanG-type vancomycin-resistant Enterococcus faecalis strains isolated in Canada. Antimicrob Agents Chemother. 2006;50:2217–2221. doi:10.1128/AAC.01541-05. 16723588 PMC1479100

[61] Abril C, Brodard I, Perreten V. Two novel antibiotic resistance genes, tet(44) and ant(6)-ib, are located within a transferable pathogenicity island in Campylobacter fetus subsp. fetus. Antimicrob Agents Chemother. 2010;54:3052–3055. doi:10.1128/AAC.00304-10. 20479200 PMC2897286

[62] Ishikawa J, Chiba K, Kurita H, Satoh H. Contribution of rpoB2 RNA polymerase beta subunit gene to rifampin resistance in Nocardia species. Antimicrob Agents Chemother. 2006;50:1342–1346. doi:10.1128/AAC.50.4.1342-1346.2006. 16569850 PMC1426977

[63] Zaw MT, Emran NA, Lin Z. Mutations inside rifampicin-resistance determining region of rpoB gene associated with rifampicin-resistance in mycobacterium tuberculosis. J Infect Public Health. 2018;11:605–610. doi:10.1016/j.jiph.2018.04.005. 29706316

[64] Kazimierczak KA, Rincon MT, Patterson AJ, Martin JC, Young P, Flint HJ, Scott KP. A new tetracycline efflux gene, tet(40), is located in tandem with tet(O/32/O) in a human gut Firmicute bacterium and in metagenomic library clones. Antimicrob Agents Chemother. 2008;52:4001–4009. doi:10.1128/AAC.00308-08. 18779355 PMC2573101

[65] Baucheron S, Imberechts H, Chaslus-Dancla E, Cloeckaert A. The AcrB multidrug transporter plays a major role in high-level fluoroquinolone resistance in Salmonella enterica serovar typhimurium phage type DT204. Microb Drug Resist. 2002;8:281–289. doi:10.1089/10766290260469543. 12523625

[66] Webber MA, Piddock LJ. Absence of mutations in marRAB or soxRS in acrB-overexpressing fluoroquinolone-resistant clinical and veterinary isolates of Escherichia coli. Antimicrob Agents Chemother. 2001;45:1550–1552. doi:10.1128/AAC.45.5.1550-1552.2001. 11302826 PMC90504

[67] Rahman T, Yarnall B, Doyle DA. Efflux drug transporters at the forefront of antimicrobial resistance. Eur Biophys J. 2017;46:647–653. doi:10.1007/s00249-017-1238-2. 28710521 PMC5599465

[68] Jiang X, Hall AB, Xavier RJ, Alm EJ, Alcaraz LD. Comprehensive analysis of chromosomal mobile genetic elements in the gut microbiome reveals phylum-level niche-adaptive gene pools. PloS One. 2019;14:e0223680. doi:10.1371/journal.pone.0223680. 31830054 PMC6907783

[69] Walpole SC, Prieto-Merino D, Edwards P, Cleland J, Stevens G, Roberts I. The weight of nations: an estimation of adult human biomass. BMC Public Health. 2012;12:439. doi:10.1186/1471-2458-12-439. 22709383 PMC3408371

[70] Nair AB, Jacob S. A simple practice guide for dose conversion between animals and human. J Basic Clin Pharm. 2016;7:27–31. doi:10.4103/0976-0105.177703. 27057123 PMC4804402

[71] Derington CG, Benavides N, Delate T, Fish DN. Multiple-dose oral Fosfomycin for treatment of complicated urinary tract infections in the outpatient setting. Open Forum Infect Dis. 2020;7:ofaa034. doi:10.1093/ofid/ofaa034. 32123690 PMC7036595

