# Supplementary material for: A metagenomic analysis for combination therapy of multiple classes of antibiotics on the prevention of the spread of antibiotic-resistant genes
Source: Gut Microbes. 2023 Oct 31;15(2):2271150. doi: 10.1080/19490976.2023.2271150 (PMC10621307; doi:10.1080/19490976.2023.2271150)
Supplement: Supplemental Material [file KGMI_A_2271150_SM4826.zip › KGMI_A_2271150-supplemental material/Supplementary Figures Antibiotic Resistance Gut Microbes Final.docx]

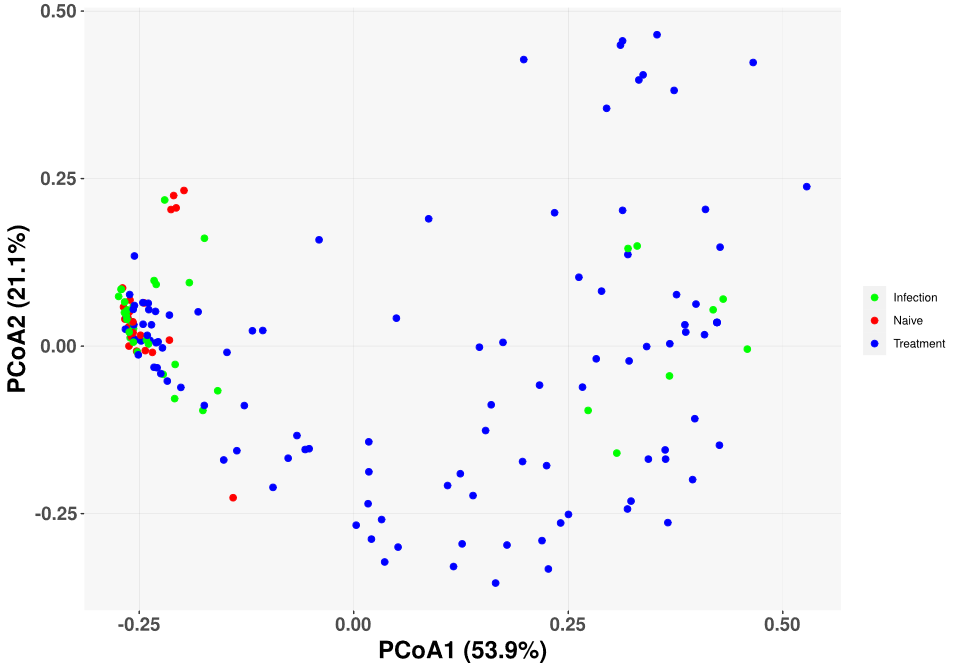


Supplementary Figure 1: Principal Coordinate Analysis (PCoaA) based on Bray–Curtis dissimilarity of ARG abundances for all sample groups across all cohorts (a) low dose Ciprofloxacin. (b) low dose Fosfomycin. (c) low dose combination Ampicillin, Ciprofloxacin. (d) high dose combination Ampicillin, Ciprofloxacin. (e) high dose combination Ampicillin, Fosfomycin. (f) high dose combination Ciprofloxacin, Fosfomycin. (g) triple combination low dose Ampicillin, Ciprofloxacin, Fosfomycin. (h) triple combination high dose Ampicillin, Ciprofloxacin, Fosfomycin.


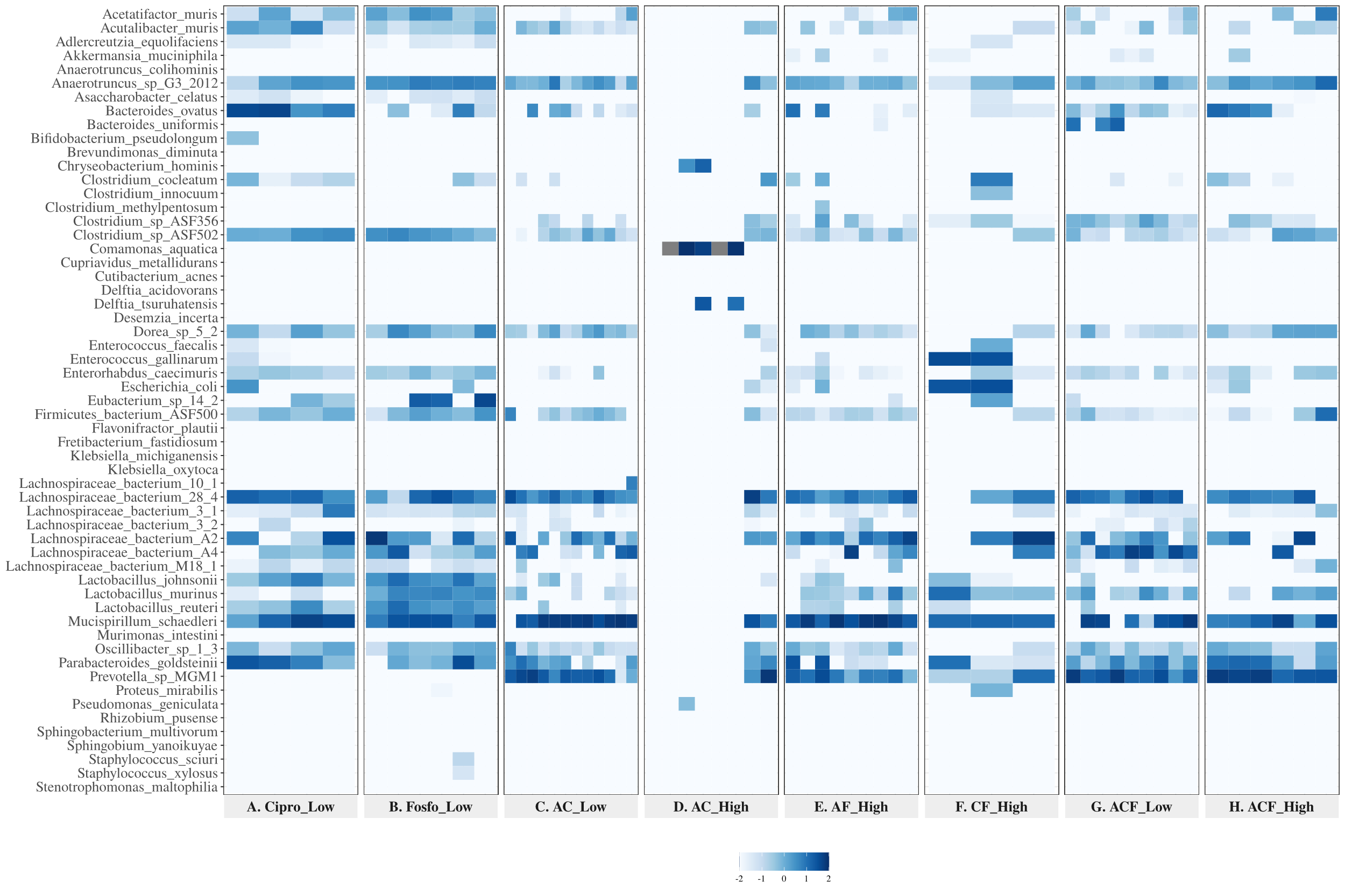
Supplementary Figure 2: Control group of gut microbiome analysis. Heatmap representing log-transformed relative abundance of the bacterial species in each control group (a) low dose Ciprofloxacin. (b) low dose Fosfomycin. (c) low dose combination Ampicillin, Ciprofloxacin. (d) high dose combination Ampicillin, Ciprofloxacin. (e) high dose combination Ampicillin, Fosfomycin. (f) high dose combination Ciprofloxacin, Fosfomycin. (g) triple combination low dose Ampicillin, Ciprofloxacin, Fosfomycin. (h) triple combination high dose Ampicillin, Ciprofloxacin, Fosfomycin. A total of 75 individual bacteria species were identified from the eight control groups.


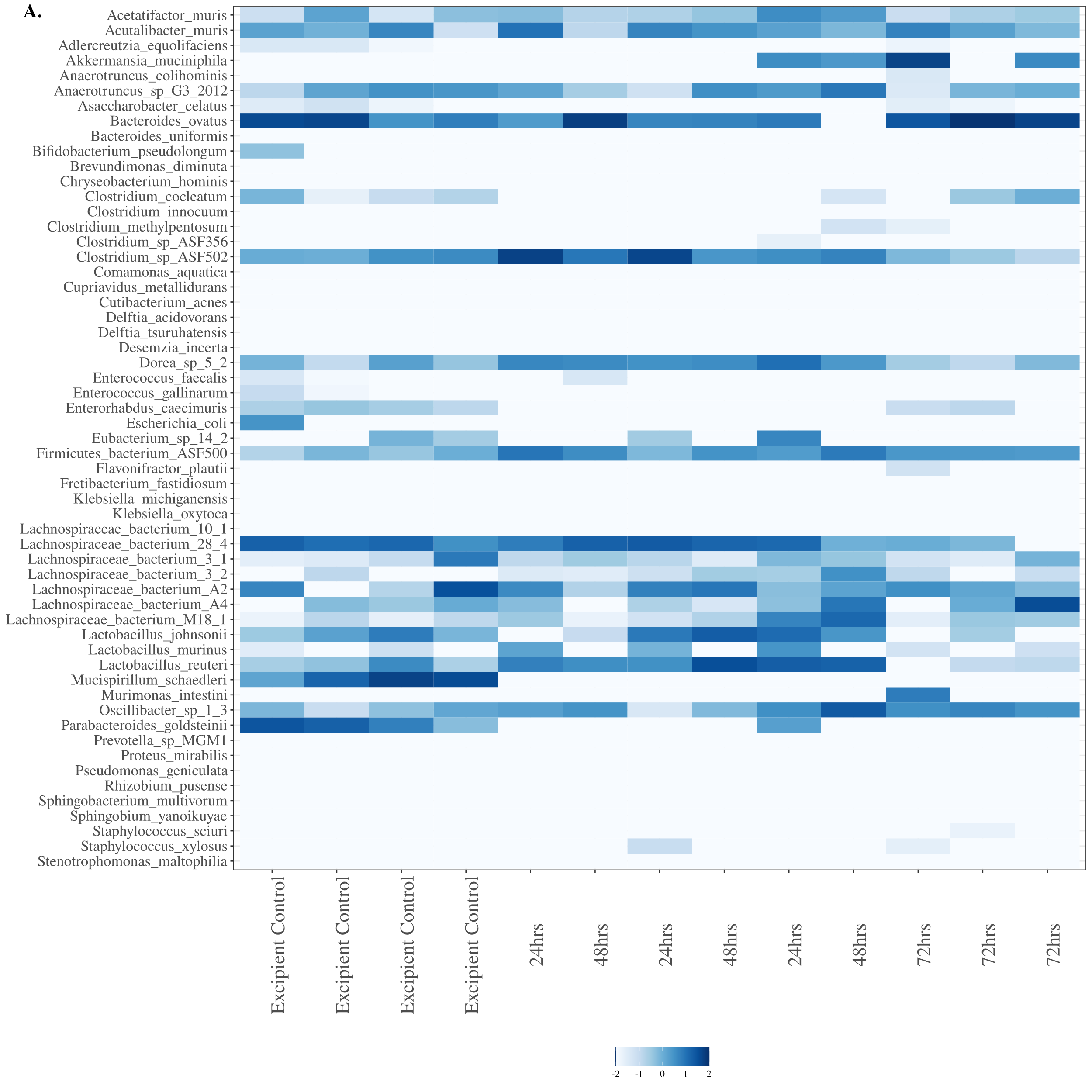

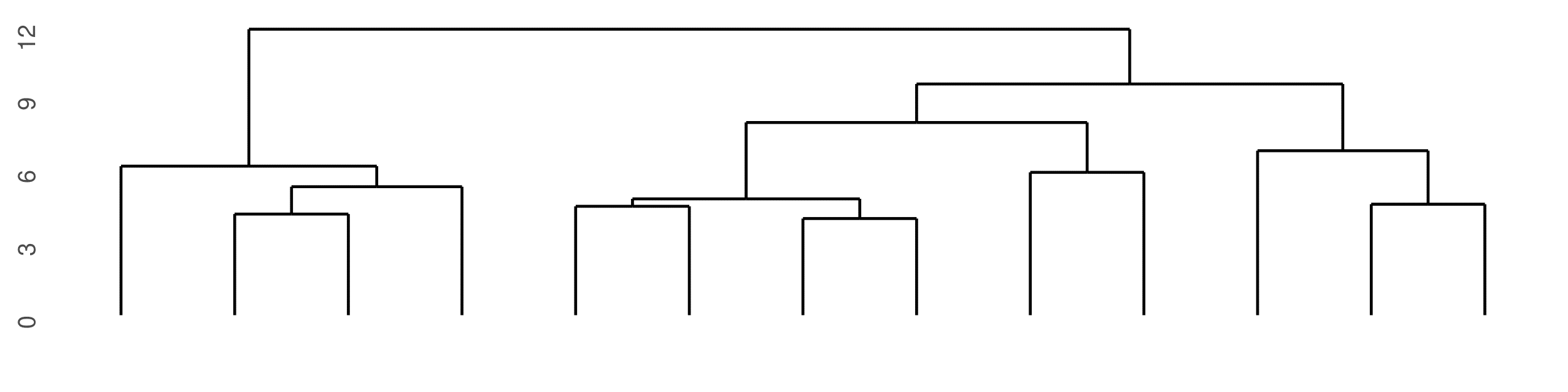

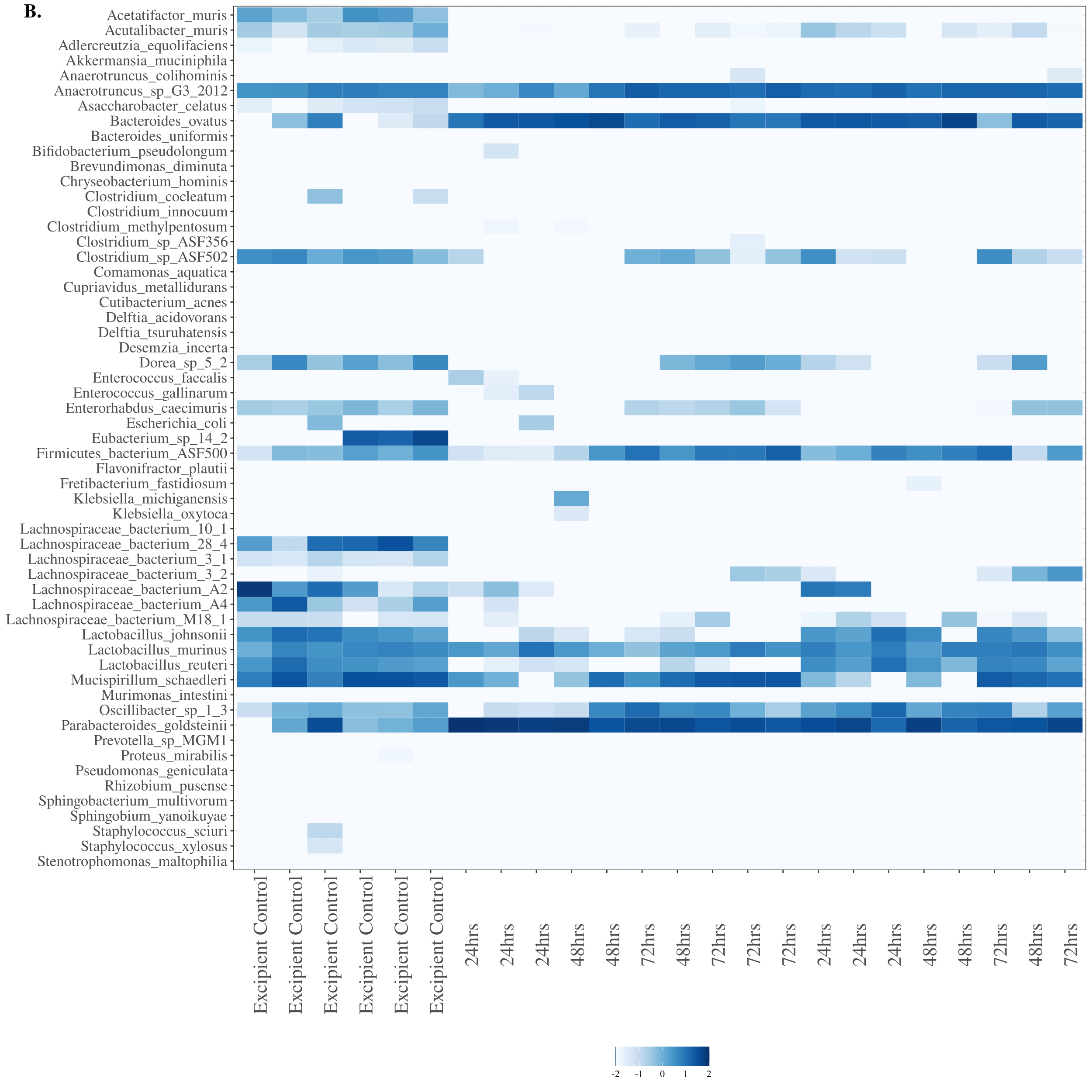

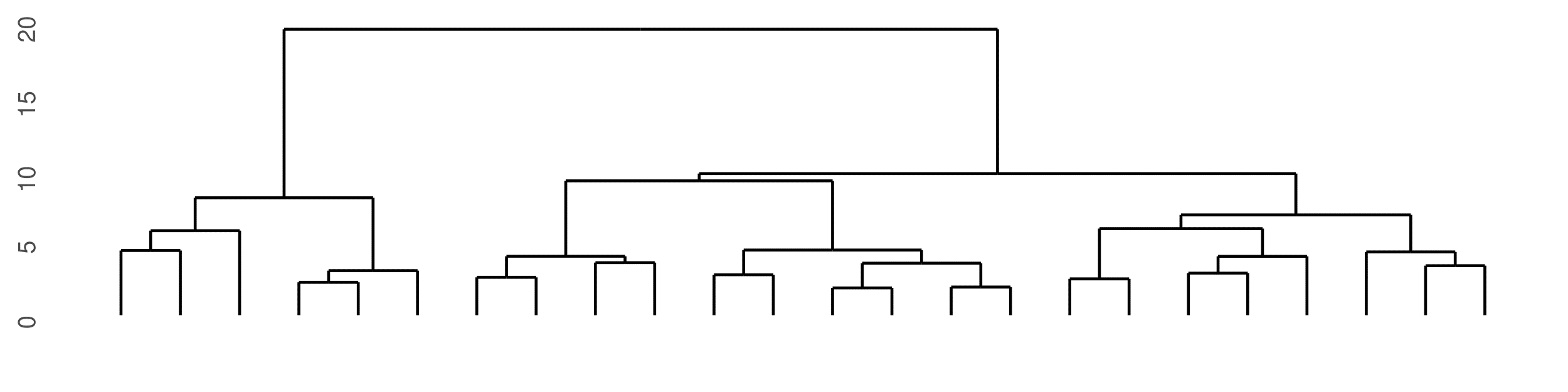

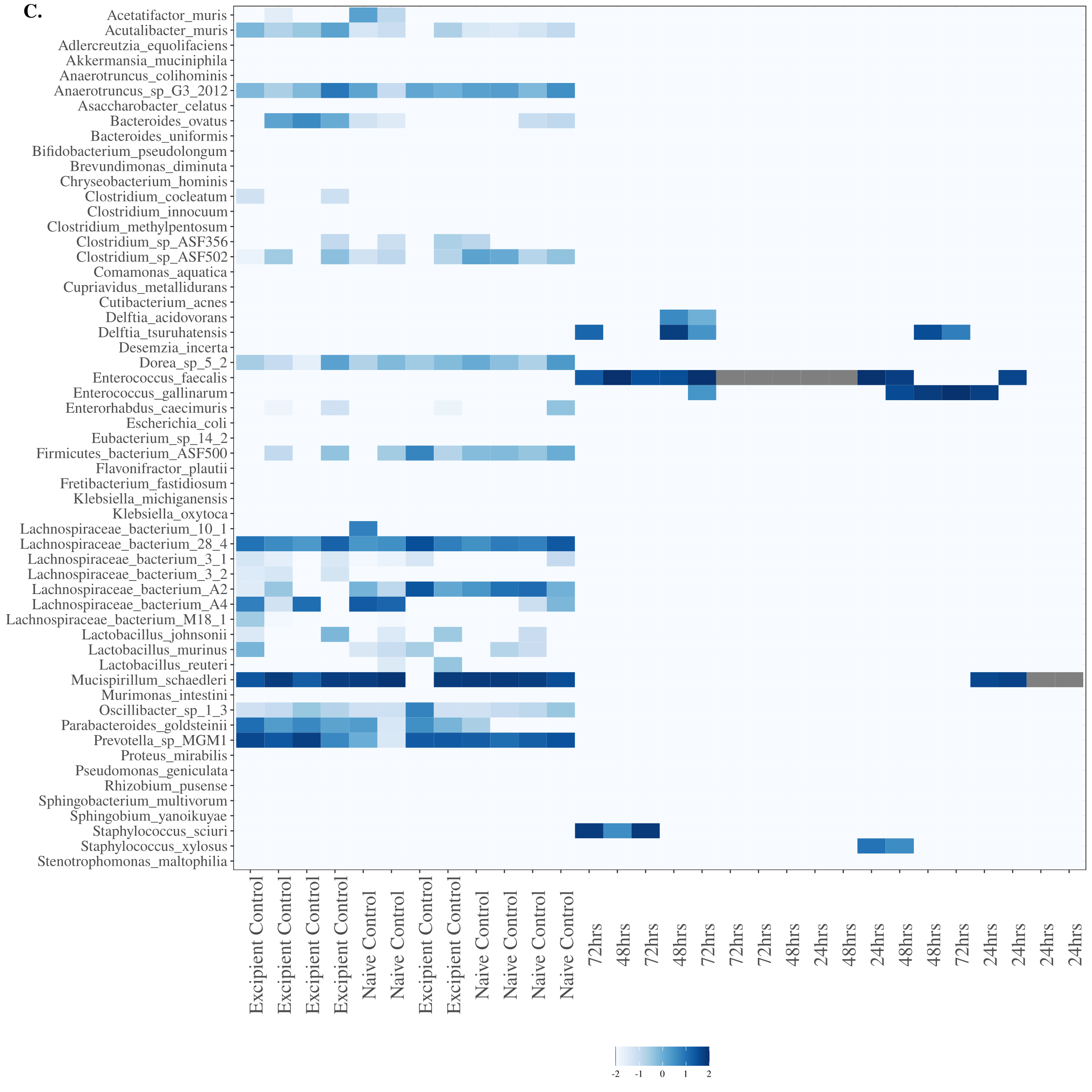

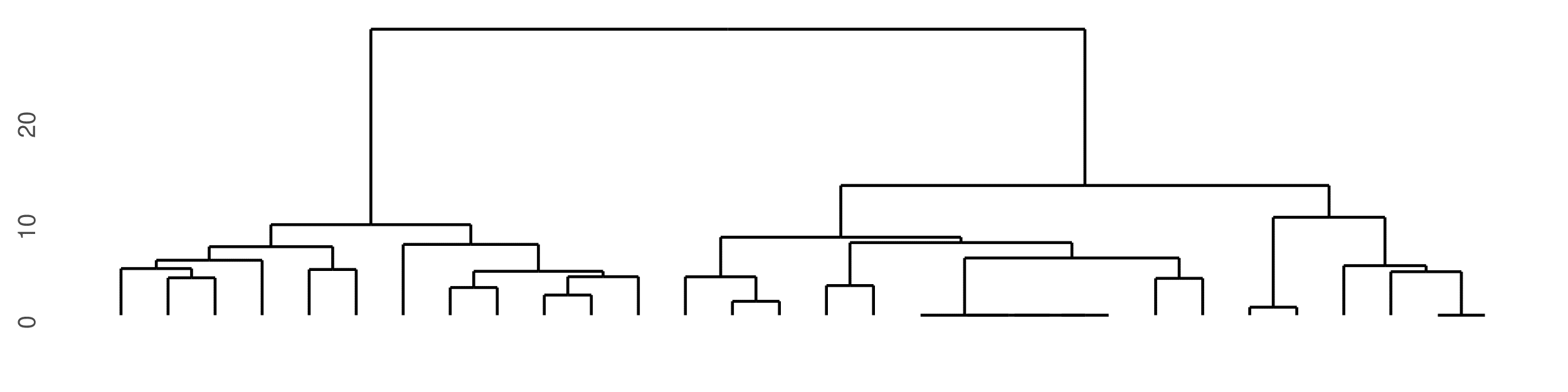

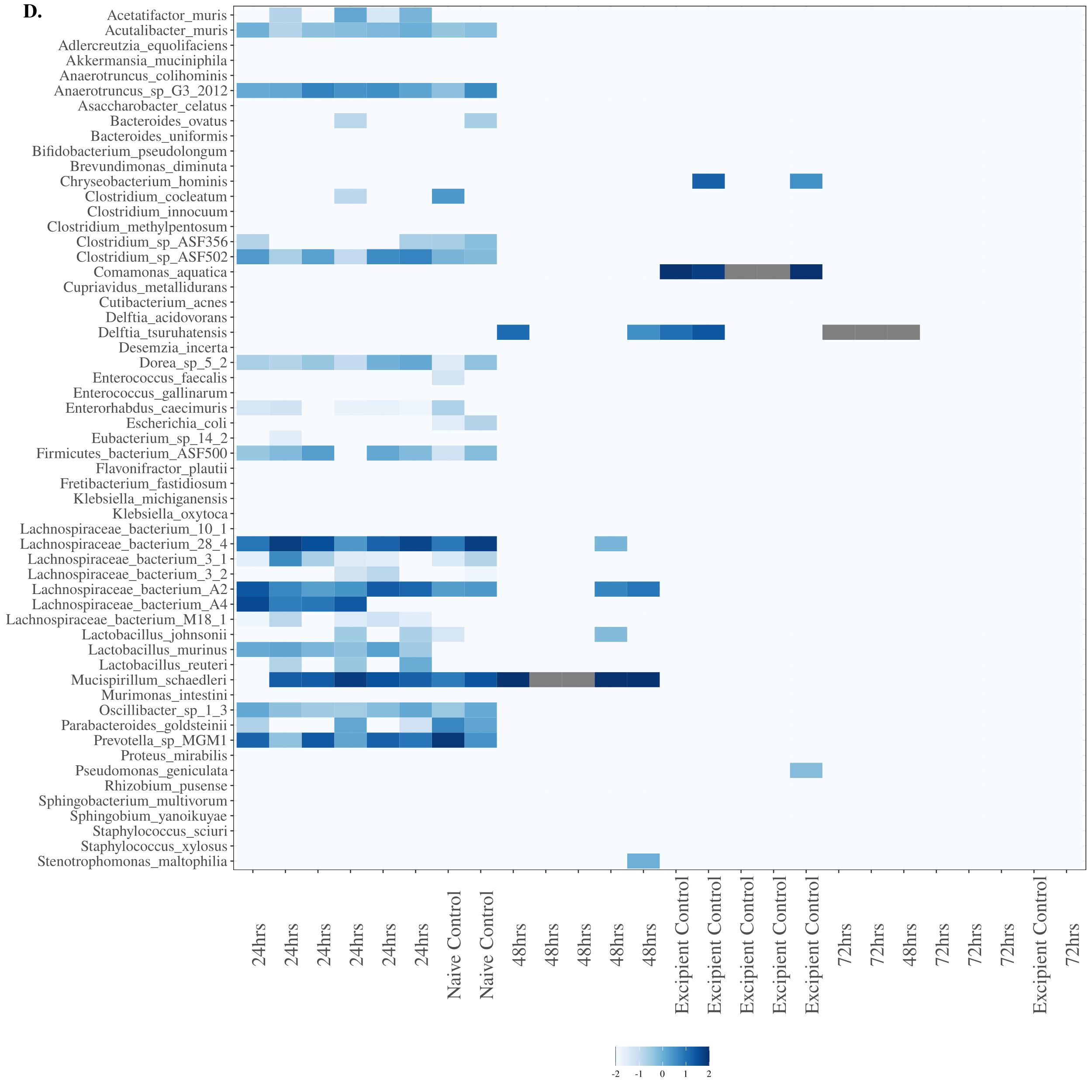

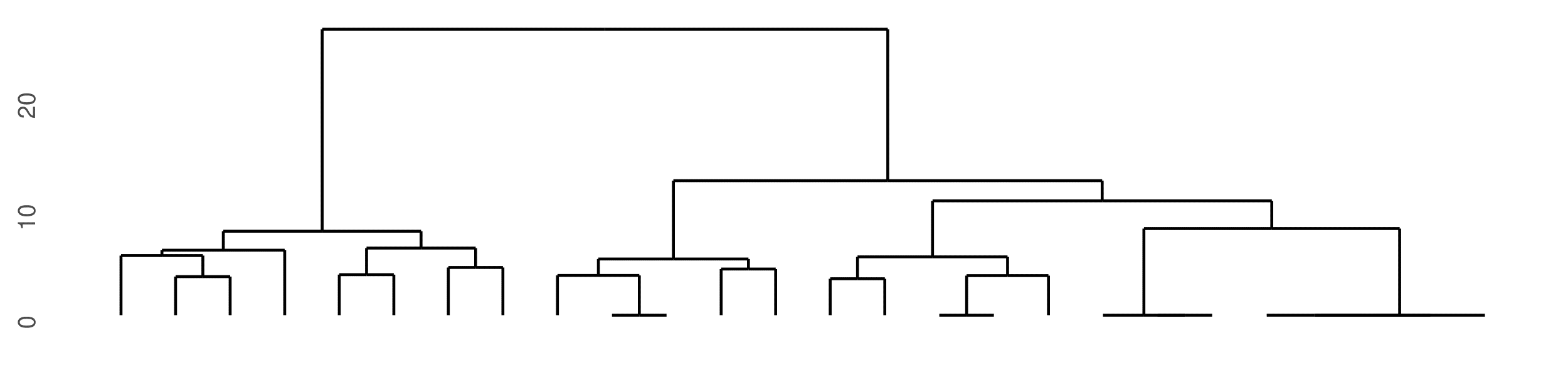

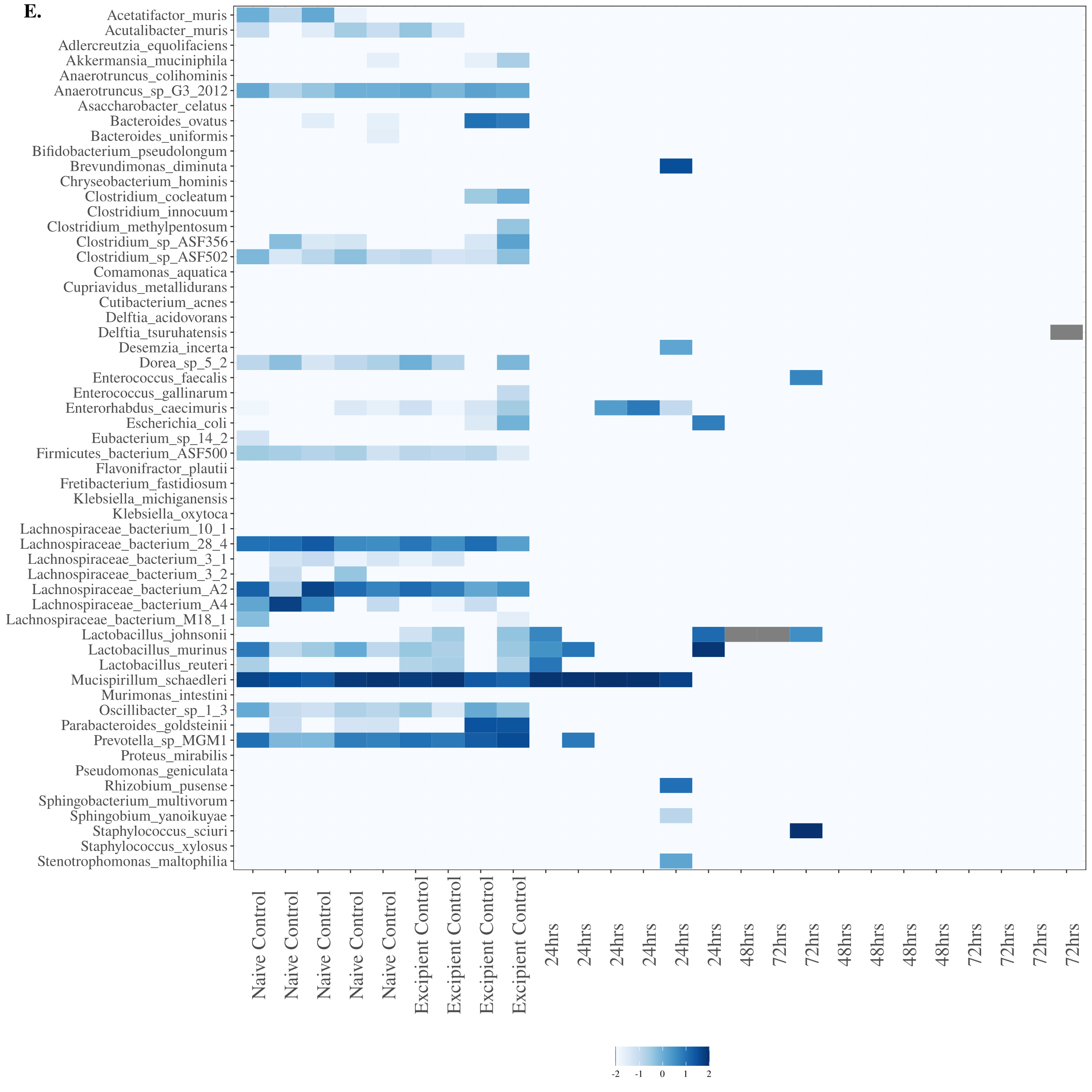

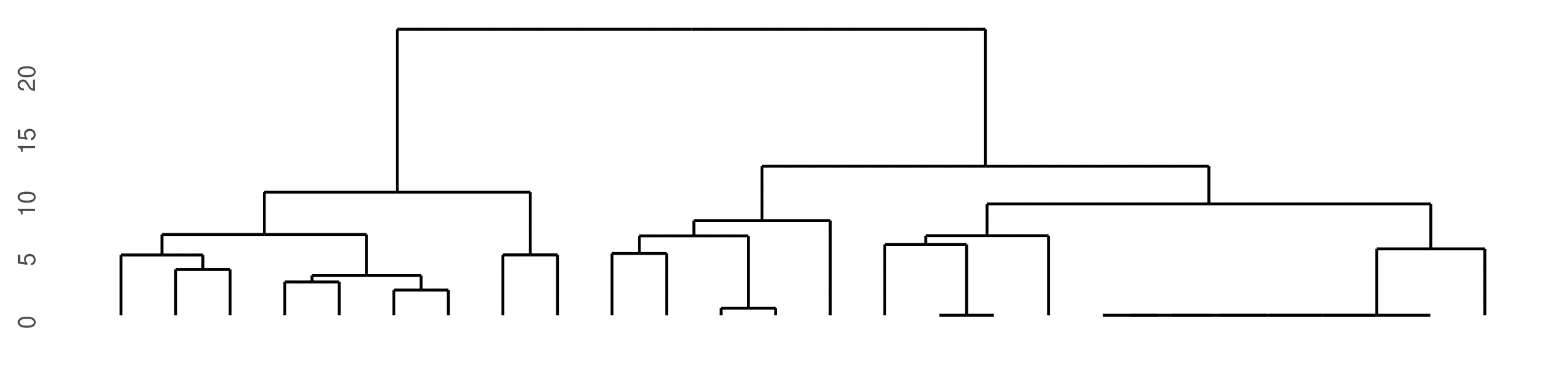

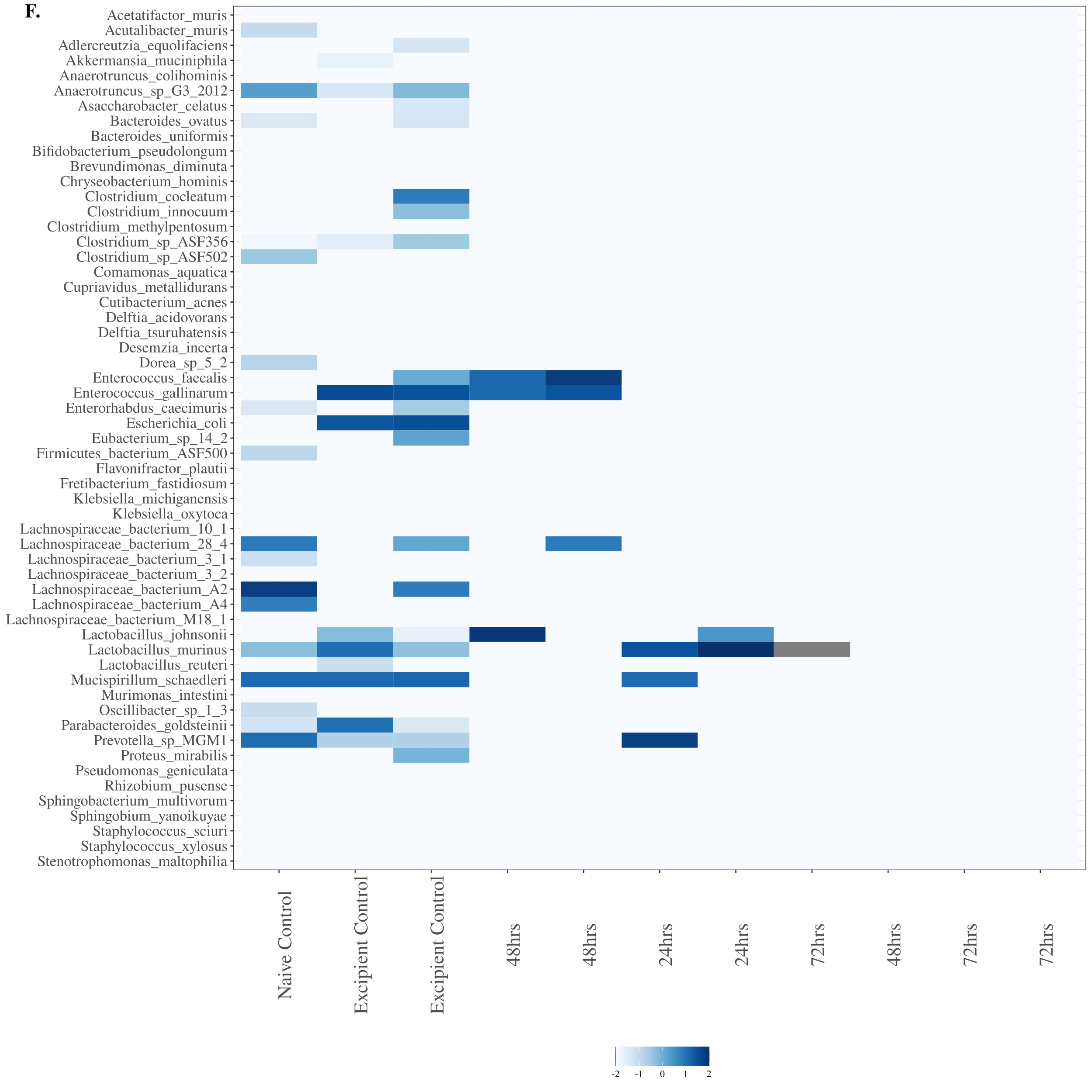

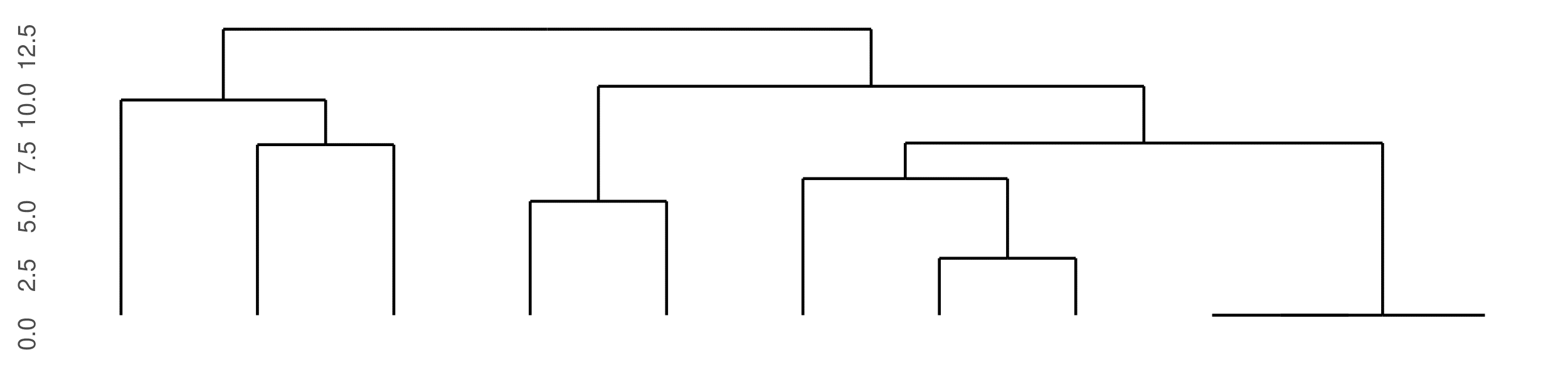

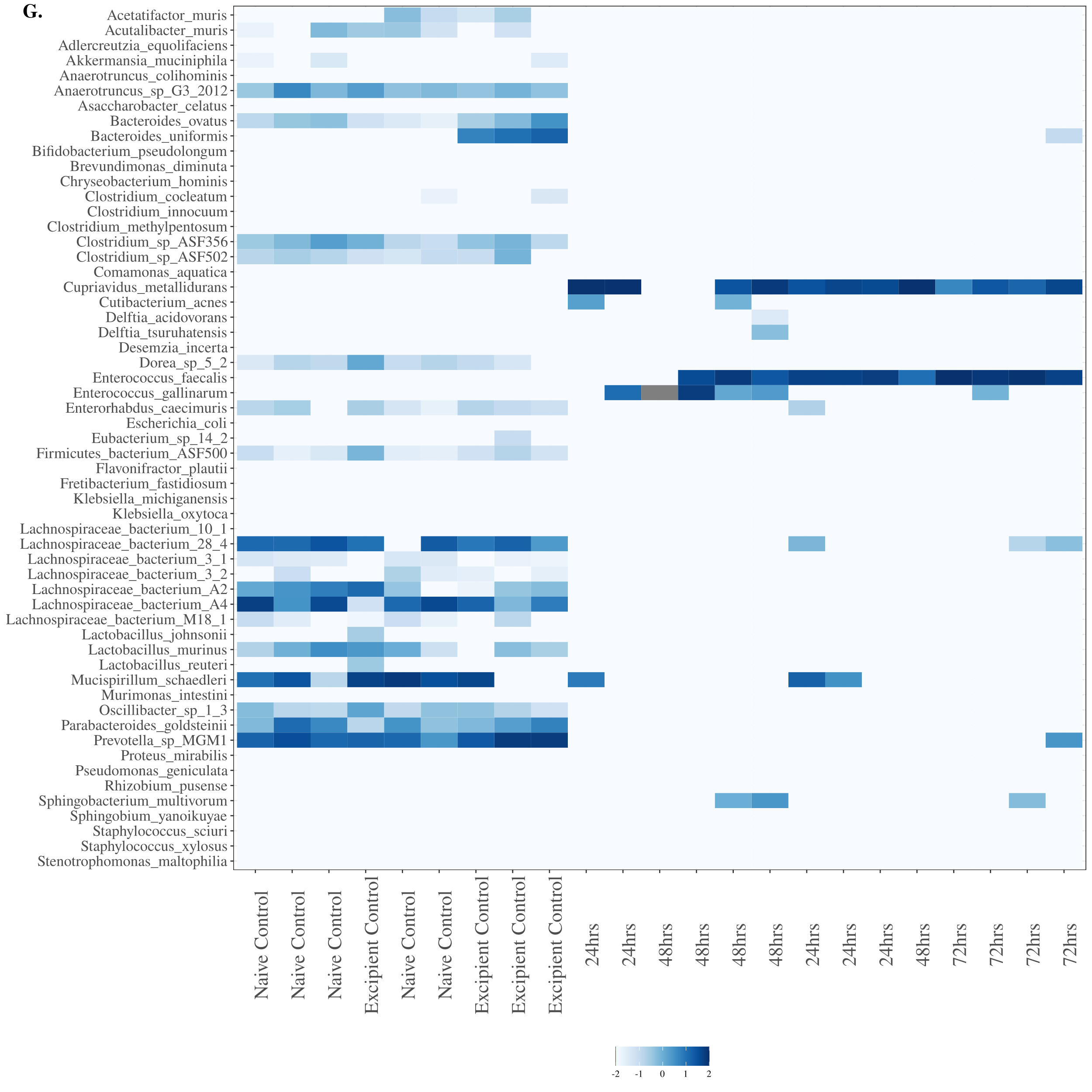

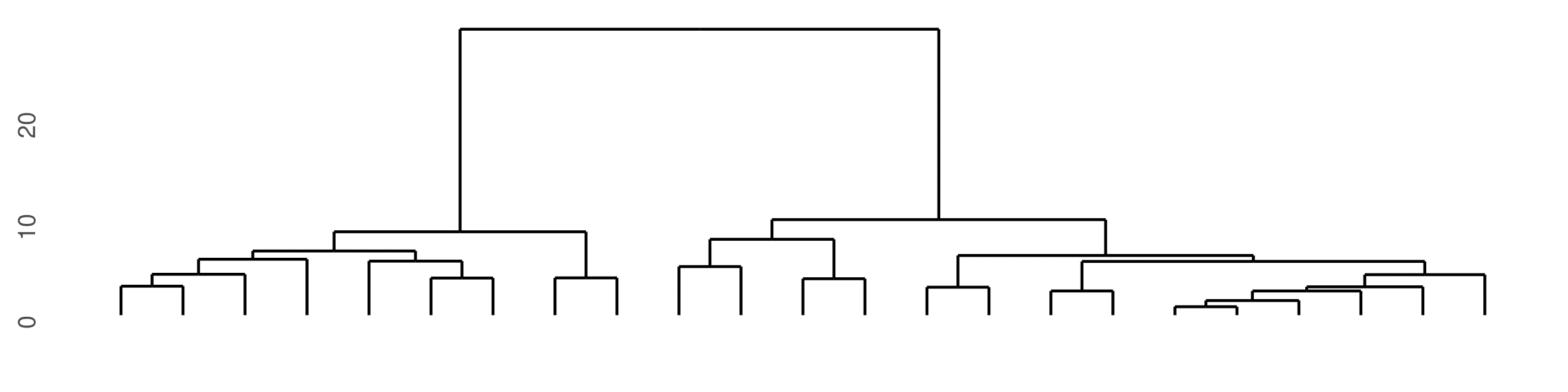

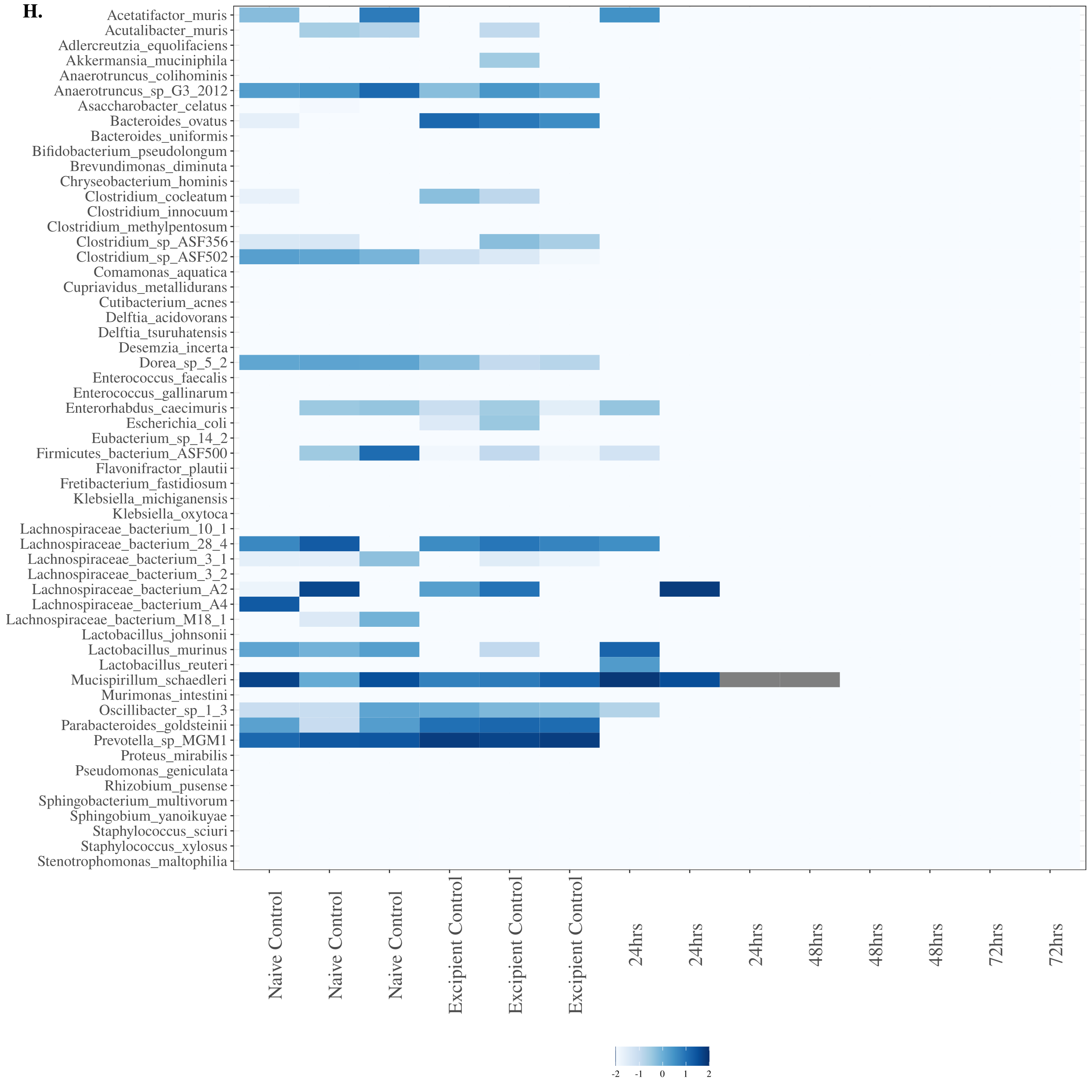

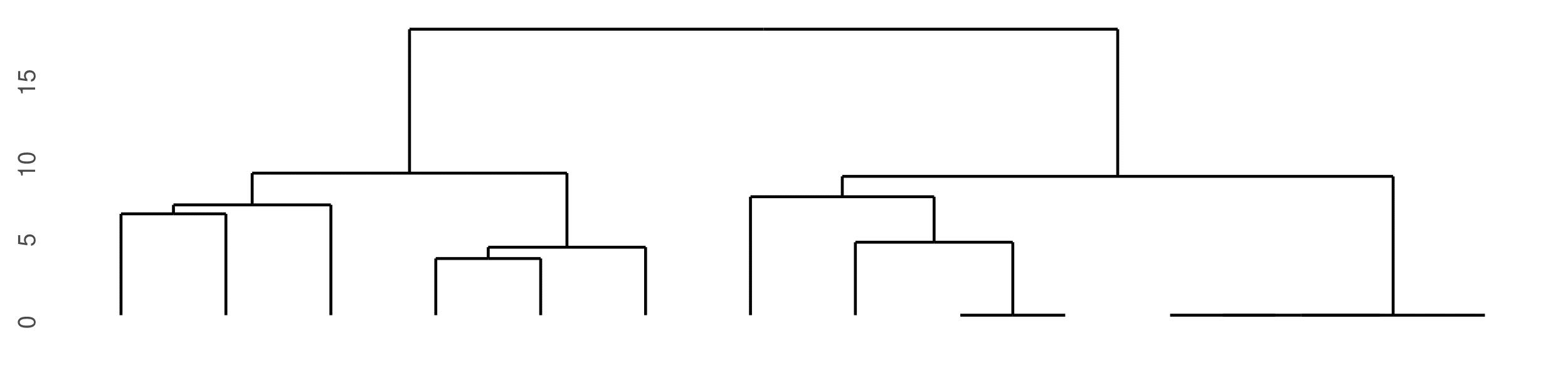


Supplementary Figure 3a-h: Heatmap with dendrogram demonstrating log-transformed relative abundance and clustering of bacterial species in the mouse gut after 24, 48, and 72 h of treatment. (a) low dose Ciprofloxacin. (b) low dose Fosfomycin. (c) low dose combination Ampicillin, Ciprofloxacin. (d) high dose combination Ampicillin, Ciprofloxacin. (e) high dose combination Ampicillin, Fosfomycin. (f) high dose combination Ciprofloxacin, Fosfomycin. (g) triple combination high dose Ampicillin, Ciprofloxacin, Fosfomycin. (h) triple combination low dose Ampicillin, Ciprofloxacin, Fosfomycin. Note the clustering together of control versus the clustering together of treated mice. Species were ordered in each graph to facilitate visualization of clustering. Color indicates the relative abundance data after log transformation.


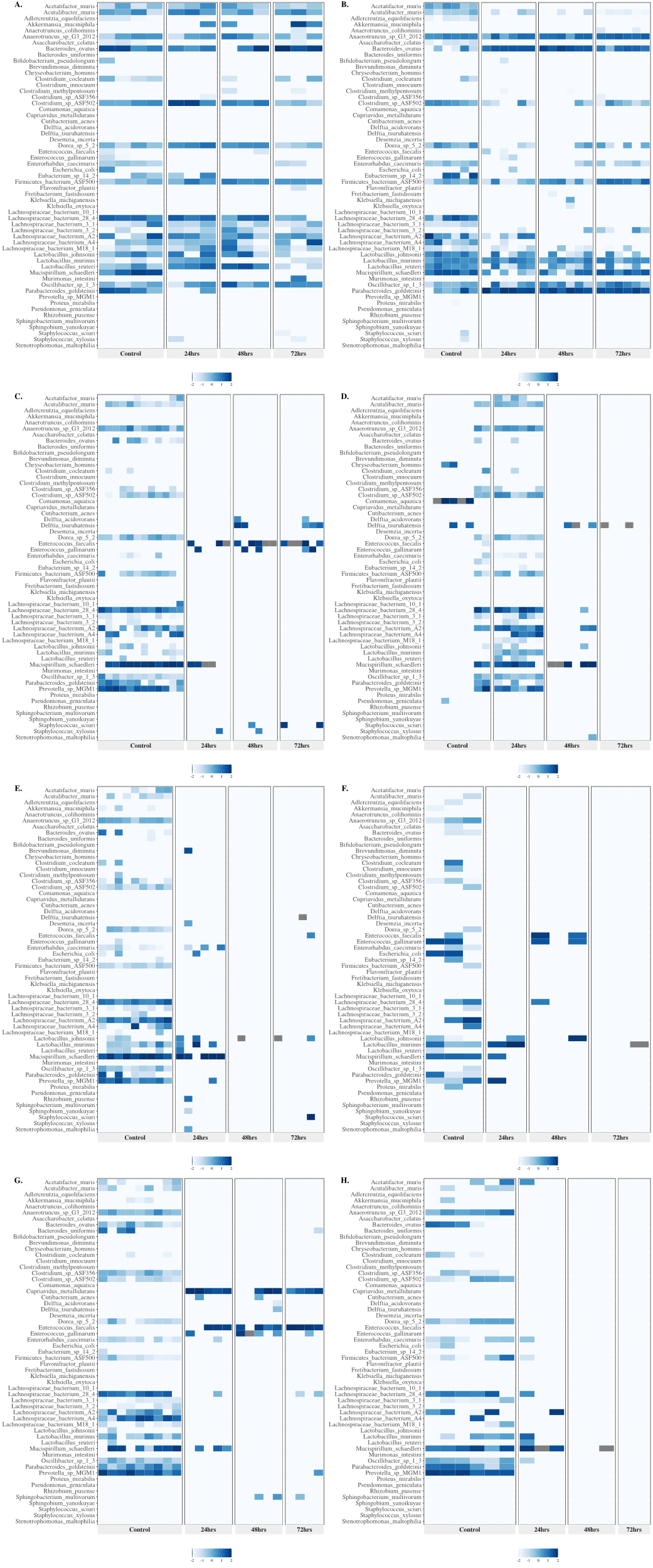
­

Supplementary Figure 4a-h: Heatmap presentation of antibiotic modulation of the log-transformed relative abundance of microbial species in the gut by different combination and monotherapy after 24, 48, and 72 h of treatment, respectively for all treatments. (a) low dose Ciprofloxacin. (b) low dose Fosfomycin. (c) low dose combination Ampicillin, Ciprofloxacin. (d) high dose combination Ampicillin, Ciprofloxacin. (e) high dose combination Ampicillin, Fosfomycin. (f) high dose combination Ciprofloxacin, Fosfomycin. (g) triple combination low dose Ampicillin, Ciprofloxacin, Fosfomycin. (h) triple combination high dose Ampicillin, Ciprofloxacin, Fosfomycin. These heatmaps represent the species listed in the same order across each heatmap to allow comparisons. Color indicates the relative abundance data after log transformation.
