# Supplementary material for: A metagenomic analysis for combination therapy of multiple classes of antibiotics on the prevention of the spread of antibiotic-resistant genes
Source: Gut Microbes. 2023 Oct 31;15(2):2271150. doi: 10.1080/19490976.2023.2271150 (PMC10621307; doi:10.1080/19490976.2023.2271150)
Supplement: Supplemental Material [file KGMI_A_2271150_SM4826.zip › KGMI_A_2271150-supplemental material/Supplementary Table 4 Mobile genetic element read count.docx]

| Supplementary Table 4: Mobile genetic element read count changes from sample treatment times compared to control samples. | | | | |
| --- | --- | --- | --- | --- |
| Ampicillin, Ciprofloxacin, Fosfomycin High Dose | Treatment | MGE | logFC | adj.P.Val |
|  | 24 h | None |  |  |
|  | 48 h | None |  |  |
|  | 72 h | None |  |  |
| Ampicillin, Ciprofloxacin, Fosfomycin Low Dose | Treatment | MGE | logFC | adj.P.Val |
|  | 24 h | None |  |  |
|  | 48 h | None |  |  |
|  | 72 h | None |  |  |
| Ampicillin, Ciprofloxacin High Dose | Treatment | MGE | logFC | adj.P.Val |
|  | 24 h | transposase | -3.8762 | 0.00015 |
|  |  | insertion_element_IS91 | -3.5387 | 5.48E-06 |
|  |  | ISCR | -2.3479 | 0.00228 |
|  | 48 h | transposase | -7.3916 | 4.25E-08 |
|  |  | insertion_element_IS91 | -5.7301 | 1.13E-06 |
|  |  | integrase | -2.2628 | 0.00569 |
|  |  | ISCR | -1.9714 | 0.03844 |
|  | 72 h | transposase | -7.7828 | 1.70E-08 |
|  |  | insertion_element_IS91 | -4.7815 | 1.82E-07 |
|  |  | integrase | -3.1591 | 0.00162 |
|  |  | ISCR | -2.4735 | 0.02166 |
| Ampicillin, Ciprofloxacin Low Dose | Treatment | MGE | logFC | adj.P.Val |
|  | 24 h | integrase | -7.5255 | 0.00623 |
|  | 48 h | Tn916 | 5.36742 | 0.00144 |
|  |  | integrase | -5.5495 | 0.02448 |
|  | 72 h | integrase | -7.529 | 0.0062 |
| Ampicillin, Fosfomycin High Dose | Treatment | MGE | logFC | adj.P.Val |
|  | 24 h | istA2 | -13.953 | 0.0323 |
|  |  | transposase | -12.572 | 8.62E-05 |
|  |  | plasmid | -9.0495 | 0.0323 |
|  | 48 h | transposase | -17.256 | 1.15E-06 |
|  |  | istA2 | -14.382 | 0.02695 |
|  |  | plasmid | -10.793 | 0.02695 |
|  | 72 h | transposase | -15.859 | 5.90E-06 |
|  |  | istA2 | -14.406 | 0.0401 |
| Ciprofloxacin, Fosfomycin High Dose | Treatment | MGE | logFC | adj.P.Val |
|  | 24 hrs | insertion_element_IS91 | -29.667 | 4.13E-06 |
|  |  | istB | -29.643 | 4.13E-06 |
|  |  | plasmid | -29.273 | 4.13E-06 |
|  |  | transposase | -25.332 | 7.16E-06 |
|  |  | integrase | -17.975 | 0.00012 |
|  |  | Tn916 | -11.571 | 0.01095 |
|  | 48 hrs | istB | -30.447 | 5.65E-06 |
|  |  | transposase | -29.562 | 5.58E-06 |
|  |  | insertion_element_IS91 | -28.705 | 5.58E-06 |
|  |  | plasmid | -27.546 | 5.58E-06 |
|  |  | integrase | -18.013 | 0.00012 |
|  |  | Tn916 | -8.6841 | 0.01586 |
|  | 72 hrs | istB | -30.495 | 5.64E-06 |
|  |  | plasmid | -29.936 | 3.38E-06 |
|  |  | insertion_element_IS91 | -29.8 | 3.38E-06 |
|  |  | transposase | -28.85 | 3.38E-06 |
|  |  | integrase | -19.274 | 0.00025 |
|  |  | istA2 | -16.157 | 0.04931 |
|  |  | Tn916 | -9.8508 | 0.01011 |
| Ciprofloxacin Low Dose | Treatment | MGE | logFC | adj.P.Val |
|  | 24 hrs | None |  |  |
|  |  | None |  |  |
|  | 72 hrs | None |  |  |
| Fosfomycin Low Dose | Treatment | MGE | logFC | adj.P.Val |
|  | 24 hrs | ISBf10 | 6.74899 | 8.58E-05 |
|  |  | plasmid | 4.26571 | 0.00895 |
|  |  | transposase | -8.1311 | 3.83E-05 |
|  |  | integrase | -7.3928 | 3.83E-05 |
|  |  | insertion_element_IS91 | -3.408 | 0.00366 |
|  |  | Tn916 | -2.6909 | 0.02757 |
|  | 48 hrs | plasmid | 7.76006 | 0.00136 |
|  |  | ISBf10 | 6.62251 | 0.00057 |
|  |  | ISCR | 1.62575 | 0.03082 |
|  |  | transposase | -9.2001 | 0.00012 |
|  |  | integrase | -7.1235 | 0.00046 |
|  |  | insertion_element_IS91 | -2.9098 | 0.02761 |
|  | 72 hrs | transposase | -8.7107 | 1.23E-06 |
|  |  | integrase | -7.4411 | 1.43E-06 |
|  |  | insertion_element_IS91 | -5.4081 | 1.43E-06 |
|  |  | Tn916 | -3.0716 | 0.00428 |
