# Supplementary material for: A metagenomic analysis for combination therapy of multiple classes of antibiotics on the prevention of the spread of antibiotic-resistant genes
Source: Gut Microbes. 2023 Oct 31;15(2):2271150. doi: 10.1080/19490976.2023.2271150 (PMC10621307; doi:10.1080/19490976.2023.2271150)
Supplement: Supplemental Material [file KGMI_A_2271150_SM4826.zip › KGMI_A_2271150-supplemental material/Supplementary Table1 Microbiome ReadCounts.docx]

| Supplementary Table 1: Sample names, cohorts, samples types, raw genomic read counts, and bacterial genomic read counts for all samples. | | | | |
| --- | --- | --- | --- | --- |
| Sample Name | cohort | Sample Type | Raw read Count | Bac Read Count |
| 25_acf_high_ncon | amp_cipro_fosfo_high | naive control | 23045595 | 12542817 |
| 27_acf_high_ncon | amp_cipro_fosfo_high | naive control | 49705290 | 26806798 |
| 29_acf_high_ncon | amp_cipro_fosfo_high | naive control | 30008281 | 18042444 |
| 19_acf_high_icon | amp_cipro_fosfo_high | excipient treatment control | 31071857 | 19887813 |
| 21_acf_high_icon | amp_cipro_fosfo_high | excipient treatment control | 25488376 | 13876041 |
| 23_acf_high_icon | amp_cipro_fosfo_high | excipient treatment control | 30295190 | 15530051 |
| 1_acf_high_24 | amp_cipro_fosfo_high | 24h | 32441913 | 1770153 |
| 3_acf_high_24 | amp_cipro_fosfo_high | 24h | 28563108 | 98301 |
| 5_acf_high_24 | amp_cipro_fosfo_high | 24h | 41394884 | 330310 |
| 7_acf_high_48 | amp_cipro_fosfo_high | 48h | 37116101 | 160933 |
| 9_acf_high_48 | amp_cipro_fosfo_high | 48h | 30639691 | 55528 |
| 11_acf_high_48 | amp_cipro_fosfo_high | 48h | 61010124 | 91986 |
| 13_acf_high_72 | amp_cipro_fosfo_high | 72h | 33477648 | 39476 |
| 15_acf_high_72 | amp_cipro_fosfo_high | 72h | 20711799 | 23699 |
| 25_acf_low_ncon | amp_cipro_fosfo_low | naive control | 32136812 | 23067939 |
| 26_acf_low_ncon | amp_cipro_fosfo_low | naive control | 33901227 | 18563370 |
| 27_acf_low_ncon | amp_cipro_fosfo_low | naive control | 36337758 | 23725392 |
| 28_acf_low_ncon | amp_cipro_fosfo_low | naive control | 38393717 | 26632005 |
| 30_acf_low_ncon | amp_cipro_fosfo_low | naive control | 39336930 | 24818207 |
| 19_acf_low_icon | amp_cipro_fosfo_low | excipient treatment control | 43110357 | 30447520 |
| 20_acf_low_icon | amp_cipro_fosfo_low | excipient treatment control | 36278089 | 25742882 |
| 23_acf_low_icon | amp_cipro_fosfo_low | excipient treatment control | 30387541 | 17623973 |
| 24_acf_low_icon | amp_cipro_fosfo_low | excipient treatment control | 37784576 | 23026473 |
| 2_acf_low_24 | amp_cipro_fosfo_low | 24h | 37469087 | 442635 |
| 3_acf_low_24 | amp_cipro_fosfo_low | 24h | 34890111 | 146416 |
| 4_acf_low_24 | amp_cipro_fosfo_low | 24h | 33131372 | 255306 |
| 5_acf_low_24 | amp_cipro_fosfo_low | 24h | 38773224 | 1057991 |
| 6_acf_low_24 | amp_cipro_fosfo_low | 24h | 36813185 | 365148 |
| 7_acf_low_48 | amp_cipro_fosfo_low | 48h | 72900416 | 662145 |
| 8_acf_low_48 | amp_cipro_fosfo_low | 48h | 35771334 | 68204 |
| 9_acf_low_48 | amp_cipro_fosfo_low | 48h | 43582316 | 727025 |
| 11_acf_low_48 | amp_cipro_fosfo_low | 48h | 46935801 | 114419 |
| 12_acf_low_48 | amp_cipro_fosfo_low | 48h | 40886599 | 209856 |
| 13_acf_low_72 | amp_cipro_fosfo_low | 72h | 40581227 | 237944 |
| 14_acf_low_72 | amp_cipro_fosfo_low | 72h | 36265347 | 202151 |
| 15_acf_low_72 | amp_cipro_fosfo_low | 72h | 33684029 | 162776 |
| 18_acf_low_72 | amp_cipro_fosfo_low | 72h | 34681357 | 482995 |
| 26_ac_high_ncon | amp_cipro_high | naive control | 33118745 | 21123915 |
| 29_ac_high_ncon | amp_cipro_high | naive control | 37840634 | 20700844 |
| 19_ac_high_icon | amp_cipro_high | excipient treatment control | 20993418 | 23246 |
| 20_ac_high_icon | amp_cipro_high | excipient treatment control | 33681306 | 105185 |
| 21_ac_high_icon | amp_cipro_high | excipient treatment control | 23030535 | 85818 |
| 22_ac_high_icon | amp_cipro_high | excipient treatment control | 34629446 | 97670 |
| 23_ac_high_icon | amp_cipro_high | excipient treatment control | 31768202 | 42477 |
| 24_ac_high_icon | amp_cipro_high | excipient treatment control | 31367860 | 66280 |
| 1_ac_high_24 | amp_cipro_high | 24h | 15937469 | 13169663 |
| 2_ac_high_24 | amp_cipro_high | 24h | 28957069 | 19604827 |
| 3_ac_high_24 | amp_cipro_high | 24h | 19642995 | 13749255 |
| 4_ac_high_24 | amp_cipro_high | 24h | 31972309 | 22135899 |
| 5_ac_high_24 | amp_cipro_high | 24h | 14895908 | 11547248 |
| 6_ac_high_24 | amp_cipro_high | 24h | 30901392 | 21293040 |
| 7_ac_high_48 | amp_cipro_high | 48h | 20392823 | 266561 |
| 8_ac_high_48 | amp_cipro_high | 48h | 36688439 | 518662 |
| 9_ac_high_48 | amp_cipro_high | 48h | 16849128 | 273966 |
| 10_ac_high_48 | amp_cipro_high | 48h | 28789172 | 152777 |
| 11_ac_high_48 | amp_cipro_high | 48h | 24844469 | 244479 |
| 12_ac_high_48 | amp_cipro_high | 48h | 28802160 | 219073 |
| 13_ac_high_72 | amp_cipro_high | 72h | 27416052 | 38677 |
| 14_ac_high_72 | amp_cipro_high | 72h | 26392332 | 44075 |
| 15_ac_high_72 | amp_cipro_high | 72h | 19617023 | 46978 |
| 16_ac_high_72 | amp_cipro_high | 72h | 28265781 | 75912 |
| 17_ac_high_72 | amp_cipro_high | 72h | 19946279 | 79405 |
| 18_ac_high_72 | amp_cipro_high | 72h | 30874166 | 121125 |
| 25_ac_low_ncon | amp_cipro_low | naive control | 25109918 | 11895049 |
| 26_ac_low_ncon | amp_cipro_low | naive control | 22928317 | 13110082 |
| 27_ac_low_ncon | amp_cipro_low | naive control | 25670619 | 16887135 |
| 28_ac_low_ncon | amp_cipro_low | naive control | 17630562 | 11708648 |
| 29_ac_low_ncon | amp_cipro_low | naive control | 30692776 | 17783034 |
| 30_ac_low_ncon | amp_cipro_low | naive control | 31798505 | 16911823 |
| 19_ac_low_icon | amp_cipro_low | excipient treatment control | 15803854 | 10501648 |
| 20_ac_low_icon | amp_cipro_low | excipient treatment control | 24320961 | 15986914 |
| 21_ac_low_icon | amp_cipro_low | excipient treatment control | 10584587 | 6346138 |
| 22_ac_low_icon | amp_cipro_low | excipient treatment control | 21801743 | 14013965 |
| 23_ac_low_icon | amp_cipro_low | excipient treatment control | 14596102 | 8444932 |
| 24_ac_low_icon | amp_cipro_low | excipient treatment control | 25215163 | 17062922 |
| 1_ac_low_24 | amp_cipro_low | 24h | 14320954 | 262216 |
| 2_ac_low_24 | amp_cipro_low | 24h | 9197853 | 32591 |
| 3_ac_low_24 | amp_cipro_low | 24h | 13839673 | 69284 |
| 4_ac_low_24 | amp_cipro_low | 24h | 9837467 | 21614 |
| 5_ac_low_24 | amp_cipro_low | 24h | 56766651 | 71390 |
| 6_ac_low_24 | amp_cipro_low | 24h | 11633297 | 56638 |
| 7_ac_low_48 | amp_cipro_low | 48h | 23967870 | 63587 |
| 8_ac_low_48 | amp_cipro_low | 48h | 13324728 | 47690 |
| 9_ac_low_48 | amp_cipro_low | 48h | 23620804 | 43352 |
| 10_ac_low_48 | amp_cipro_low | 48h | 10560301 | 11935 |
| 11_ac_low_48 | amp_cipro_low | 48h | 39009166 | 36224 |
| 12_ac_low_48 | amp_cipro_low | 48h | 16653656 | 26385 |
| 13_ac_low_72 | amp_cipro_low | 72h | 19146035 | 36593 |
| 14_ac_low_72 | amp_cipro_low | 72h | 18434568 | 127336 |
| 15_ac_low_72 | amp_cipro_low | 72h | 26350408 | 48142 |
| 16_ac_low_72 | amp_cipro_low | 72h | 15716941 | 80373 |
| 17_ac_low_72 | amp_cipro_low | 72h | 16394205 | 19243 |
| 18_ac_low_72 | amp_cipro_low | 72h | 16350029 | 79978 |
| 25_af_high_ncon | amp_fosfo_high | naive control | 28198151 | 18826256 |
| 25_af_high_ncon_2 | amp_fosfo_high | naive control | 33981145 | 17803802 |
| 28_af_high_ncon | amp_fosfo_high | naive control | 37574545 | 24806425 |
| 29_af_high_ncon | amp_fosfo_high | naive control | 29068501 | 15936218 |
| 29_af_high_ncon_2 | amp_fosfo_high | naive control | 32724032 | 23404506 |
| 19_af_high_icon | amp_fosfo_high | excipient treatment control | 30453033 | 15673905 |
| 20_af_high_icon | amp_fosfo_high | excipient treatment control | 46061644 | 25540464 |
| 21_af_high_icon | amp_fosfo_high | excipient treatment control | 26850930 | 15826929 |
| 22_af_high_icon | amp_fosfo_high | excipient treatment control | 31218189 | 17878623 |
| 2_af_high_24 | amp_fosfo_high | 24h | 33888699 | 318665 |
| 2_af_high_24_2 | amp_fosfo_high | 24h | 30327928 | 7744944 |
| 3_af_high_24 | amp_fosfo_high | 24h | 40348700 | 84736 |
| 4_af_high_24 | amp_fosfo_high | 24h | 29283864 | 3058974 |
| 5_af_high_24 | amp_fosfo_high | 24h | 28629752 | 131277 |
| 6_af_high_24 | amp_fosfo_high | 24h | 27934347 | 7370470 |
| 7_af_high_48 | amp_fosfo_high | 48h | 30125597 | 79982 |
| 8_af_high_48 | amp_fosfo_high | 48h | 38673504 | 149823 |
| 10_af_high_48 | amp_fosfo_high | 48h | 30054655 | 67485 |
| 11_af_high_48 | amp_fosfo_high | 48h | 30345041 | 68211 |
| 12_af_high_48 | amp_fosfo_high | 48h | 28019519 | 485871 |
| 13_af_high_72 | amp_fosfo_high | 72h | 33610798 | 49404 |
| 14_af_high_72 | amp_fosfo_high | 72h | 33956777 | 44342 |
| 15_af_high_72 | amp_fosfo_high | 72h | 34457781 | 135261 |
| 16_af_high_72 | amp_fosfo_high | 72h | 36855647 | 103372 |
| 17_af_high_72 | amp_fosfo_high | 72h | 33235604 | 112311 |
| 18_af_high_72 | amp_fosfo_high | 72h | 34030803 | 74726 |
| 30_cf_high_ncon | cipro_fosfo_high | naive control | 38239225 | 22838186 |
| 20_cf_high_icon | cipro_fosfo_high | excipient treatment control | 34812849 | 5260319 |
| 24_cf_high_icon | cipro_fosfo_high | excipient treatment control | 40346076 | 9146560 |
| 1_cf_high_24 | cipro_fosfo_high | 24h | 47689970 | 268453 |
| 3_cf_high_24 | cipro_fosfo_high | 24h | 39074501 | 113193 |
| 8_cf_high_48 | cipro_fosfo_high | 48h | 35975717 | 134999 |
| 10_cf_high_48 | cipro_fosfo_high | 48h | 32406870 | 354603 |
| 12_cf_high_48 | cipro_fosfo_high | 48h | 39036639 | 151655 |
| 14_cf_high_72 | cipro_fosfo_high | 72h | 35838610 | 77901 |
| 16_cf_high_72 | cipro_fosfo_high | 72h | 47450737 | 78855 |
| 18_cf_high_72 | cipro_fosfo_high | 72h | 39723260 | 247379 |
| 19_cipro_low_icon | cipro_low | excipient treatment control | 31268580 | 23040907 |
| 20_cipro_low_icon | cipro_low | excipient treatment control | 30364440 | 22524590 |
| 22_cipro_low_icon | cipro_low | excipient treatment control | 34454933 | 27246120 |
| 24_cipro_low_icon | cipro_low | excipient treatment control | 19560789 | 15116905 |
| 3_cipro_low_24 | cipro_low | 24h | 26379008 | 19802429 |
| 4_cipro_low_24 | cipro_low | 24h | 28657855 | 22488698 |
| 5_cipro_low_24 | cipro_low | 24h | 34084518 | 23819226 |
| 8_cipro_low_48 | cipro_low | 48h | 35675109 | 26341655 |
| 11_cipro_low_48 | cipro_low | 48h | 26383191 | 21221036 |
| 12_cipro_low_48 | cipro_low | 48h | 30180737 | 22461778 |
| 15_cipro_low_72 | cipro_low | 72h | 33663804 | 24819964 |
| 16_cipro_low_72 | cipro_low | 72h | 35286759 | 24991543 |
| 18_cipro_low_72 | cipro_low | 72h | 32434496 | 23507810 |
| 19_fosfo_low_icon | fosfo_low | excipient treatment control | 32744288 | 24632373 |
| 20_fosfo_low_icon | fosfo_low | excipient treatment control | 20796859 | 16616507 |
| 21_fosfo_low_icon | fosfo_low | excipient treatment control | 29809325 | 23060535 |
| 22_fosfo_low_icon | fosfo_low | excipient treatment control | 31671782 | 24247023 |
| 23_fosfo_low_icon | fosfo_low | excipient treatment control | 51870563 | 36089757 |
| 24_fosfo_low_icon | fosfo_low | excipient treatment control | 38018625 | 28018993 |
| 1_fosfo_low_24 | fosfo_low | 24h | 55779809 | 26192237 |
| 2_fosfo_low_24 | fosfo_low | 24h | 28181796 | 16636127 |
| 3_fosfo_low_24 | fosfo_low | 24h | 52184879 | 27006184 |
| 4_fosfo_low_24 | fosfo_low | 24h | 36067743 | 19109102 |
| 5_fosfo_low_24 | fosfo_low | 24h | 44200656 | 23133777 |
| 6_fosfo_low_24 | fosfo_low | 24h | 30374176 | 16006124 |
| 7_fosfo_low_48 | fosfo_low | 48h | 19560537 | 10763791 |
| 8_fosfo_low_48 | fosfo_low | 48h | 22016240 | 12250422 |
| 9_fosfo_low_48 | fosfo_low | 48h | 31107415 | 18088081 |
| 10_fosfo_low_48 | fosfo_low | 48h | 19577463 | 11265634 |
| 11_fosfo_low_48 | fosfo_low | 48h | 27285236 | 14946337 |
| 12_fosfo_low_48 | fosfo_low | 48h | 15822988 | 9644263 |
| 13_fosfo_low_72 | fosfo_low | 72h | 55199794 | 42224789 |
| 14_fosfo_low_72 | fosfo_low | 72h | 26352496 | 22618556 |
| 15_fosfo_low_72 | fosfo_low | 72h | 40195008 | 34716178 |
| 16_fosfo_low_72 | fosfo_low | 72h | 36444559 | 31023260 |
| 17_fosfo_low_72 | fosfo_low | 72h | 51913701 | 42433331 |
| 18_fosfo_low_72 | fosfo_low | 72h | 31838976 | 19139931 |
