# Supplementary material for: A metagenomic analysis for combination therapy of multiple classes of antibiotics on the prevention of the spread of antibiotic-resistant genes
Source: Gut Microbes. 2023 Oct 31;15(2):2271150. doi: 10.1080/19490976.2023.2271150 (PMC10621307; doi:10.1080/19490976.2023.2271150)
Supplement: Supplemental Material [file KGMI_A_2271150_SM4826.zip › KGMI_A_2271150-supplemental material/Supplementary Table2 Microbiome Sig Populations.docx]

| Supplementary Table 2: A-H Statistically significant changes to species after oral treatment with A) Ampicillin, Ciprofloxacin, Fosfomycin at High Dose, B) Ampicillin, Ciprofloxacin, Fosfomycin at Low Dose, C) Ampicillin, Ciprofloxacin at High Dose, D) Ampicillin, Ciprofloxacin at Low Dose, E) Ampicillin, Fosfomycin at High Dose F) Ciprofloxacin, Fosfomycin at High Dose, G) Ciprofloxacin at Low Dose, and H) Fosfomycin at Low Dose | | |
| --- | --- | --- |
| **A. Ampicillin, Ciprofloxacin, Fosfomycin High Dose** | | |
| Treatment | Species | Increase/Decrease |
| 24h | Mucispirillum_schaedleri | **+** |
|  | Lactobacillus_murinus | **+** |
|  | Prevotella_sp_MGM1 | **-** |
| 48h | Mucispirillum_schaedleri | **+** |
|  | Prevotella_sp_MGM1 | **-** |
| 72h | None |  |
| **B. Ampicillin, Ciprofloxacin, Fosfomycin Low Dose** | | |
| Treatment | Species | Increase/Decrease |
| 24h | Cupriavidus_metallidurans | **+** |
|  | Enterococcus_faecalis | **+** |
|  | Cutibacterium_acnes | **+** |
|  | Prevotella_sp_MGM1 | **-** |
|  | Lachnospiraceae_bacterium_A4 | **-** |
| 48h | Cupriavidus_metallidurans | **+** |
|  | Enterococcus_gallinarum | **+** |
|  | Enterococcus_faecalis | **+** |
|  | Prevotella_sp_MGM1 | **-** |
|  | Lachnospiraceae_bacterium_A4 | **-** |
| 72h | Enterococcus_faecalis | **+** |
|  | Cupriavidus_metallidurans | **+** |
| **C. Ampicillin, Ciprofloxacin High Dose** | | |
| Treatment | Species | Increase/Decrease |
| 24h | None |  |
| 48h | Mucispirillum_schaedleri | **+** |
|  | Lachnospiraceae_bacterium_A2 | **+** |
|  | Comamonas_aquatica | **-** |
| 72h | None |  |
| **D. Ampicillin, Ciprofloxacin Low Dose** | | |
| Treatment | Species | Increase/Decrease |
| 24h | Enterococcus_faecalis | **+** |
|  | Enterococcus_gallinarum | **+** |
|  | Mucispirillum_schaedleri | **+** |
|  | Staphylococcus_xylosus | **+** |
|  | Prevotella_sp_MGM1 | **-** |
|  | Lachnospiraceae_bacterium_28_4 | **-** |
| 48h | Enterococcus_faecalis | **+** |
|  | Delftia_tsuruhatensis | **+** |
|  | Enterococcus_gallinarum | **+** |
|  | Delftia_acidovorans | **+** |
|  | Staphylococcus_sciuri | **+** |
|  | Staphylococcus_xylosus | **+** |
|  | Mucispirillum_schaedleri | **-** |
|  | Prevotella_sp_MGM1 | **-** |
|  | Lachnospiraceae_bacterium_28_4 | **-** |
| 72h | Enterococcus_faecalis | **+** |
|  | Enterococcus_gallinarum | **+** |
|  | Staphylococcus_sciuri | **+** |
|  | Delftia_tsuruhatensis | **+** |
|  | Mucispirillum_schaedleri | **-** |
|  | Prevotella_sp_MGM1 | **-** |
|  | Lachnospiraceae_bacterium_28_4 | **-** |
| **E. Ampicillin, Fosfomycin High Dose** | | |
| Treatment | Species | Increase/Decrease |
| 24h | Lactobacillus_murinus | **+** |
|  | Lactobacillus_johnsonii | **+** |
|  | Escherichia_coli | **+** |
|  | Mucispirillum_schaedleri | **+** |
|  | Lactobacillus_reuteri | **+** |
| 48h | Lactobacillus_johnsonii | **+** |
|  | Mucispirillum_schaedleri | **-** |
| 72h | Lactobacillus_johnsonii | **+** |
|  | Mucispirillum_schaedleri | **-** |
| **F. Ciprofloxacin, Fosfomycin High Dose** | | |
| Treatment | Species | Increase/Decrease |
| 24h | None |  |
| 48h | None |  |
| 72h | None |  |
| **G. Ciprofloxacin Low Dose** | | |
| Treatment | Species | Increase/Decrease |
| 24h | None |  |
| 48h | None |  |
| 72h | None |  |
| **H. Fosfomycin Low Dose** | | |
| Treatment | Species | Increase/Decrease |
| 24h | Parabacteroides_goldsteinii | **+** |
| 48h | Bacteroides_ovatus | **+** |
|  | Parabacteroides_goldsteinii | **+** |
| 72h | None |  |
